# Nanoformulation-by-design: an experimental and molecular dynamics study for polymer coated drug nanoparticles†

**DOI:** 10.1039/d0ra00408a

**Published:** 2020-05-21

**Authors:** Ioanna Danai Styliari, Vincenzo Taresco, Andrew Theophilus, Cameron Alexander, Martin Garnett, Charles Laughton

**Affiliations:** School of Pharmacy, University of Nottingham Nottingham NG7 2RD UK; GlaxoSmithKline R&D Gunnels Wood Rd Stevenage SG1 2NY UK

## Abstract

The formulation of drug compounds into nanoparticles has many potential advantages in enhancing bioavailability and improving therapeutic efficacy. However, few drug molecules will assemble into stable, well-defined nanoparticulate structures. Amphiphilic polymer coatings are able to stabilise nanoparticles, imparting defined surface properties for many possible drug delivery applications. In the present article we explore, both experimentally and *in silico*, a potential methodology to coat drug nanoparticles with an amphiphilic co-polymer. Monomethoxy polyethylene glycol–polycaprolactone (mPEG-*b*-PCL) diblock copolymers with different mPEG lengths (*M*_w_ 350, 550, 750 and 2000), designed to give different levels of colloidal stability, were used to coat the surface of indomethacin nanoparticles. Polymer coating was achieved by a flow nanoprecipitation method that demonstrated excellent batch-to-batch reproducibility and resulted in nanoparticles with high drug loadings (up to 78%). At the same time, in order to understand this modified nanoprecipitation method at an atomistic level, large-scale all-atom molecular dynamics simulations were performed in parallel using the GROMOS53a6 forcefield parameters. It was observed that the mPEG-*b*-PCL chains act synergistically with the acetone molecules to dissolve the indomethacin nanoparticle while after the removal of the acetone molecules (mimicking the evaporation of the organic solvent) a polymer–drug nanoparticle was formed (yield 99%). This work could facilitate the development of more efficient methodologies for producing nanoparticles of hydrophobic drugs coated with amphiphilic polymers. The atomistic insight from the MD simulations in tandem with the data from the drug encapsulation experiments thus leads the way to a *nanoformulation-by-design* approach for therapeutic nanoparticles.

## Abstract 2

Experimental studies of drug–polymer nanoparticle formation combined with molecular dynamics simulations provide atomistic explanations for the high drug loadings obtained.

## Graphical abstract

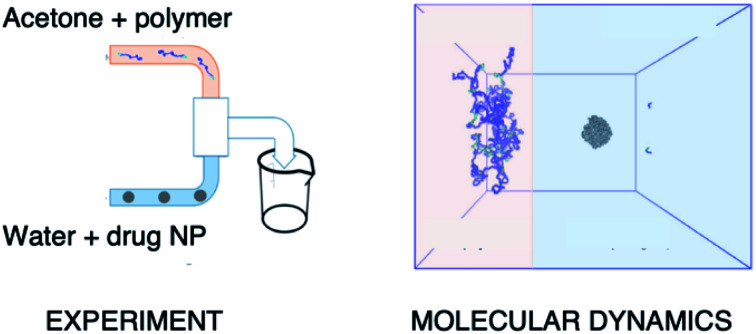

## Introduction

An ideal drug delivery system (DDS) is non-toxic, biodegradable and biocompatible,^1^ it should be able to encapsulate sufficient amounts of drug to provide a therapeutic action, which can be released at its target site.^2^ From a practical perspective, it has to be cheap, easy to manufacture, and stable prior to its administration. One of the main DDSs that have attracted a lot of interest in the pharmaceutical field are nanoparticles (NPs). NPs, usually in the range of 10–500 nm in diameter, can be modified according to their physical properties, their composition, surface chemistry and target ligands for specificity.^3^ This flexibility allows the design of sophisticated, cost-friendly and multifunctional NPs. Nanoparticulate DDSs have many potential applications in a wide range of formulations including targeted delivery of hydrophobic drugs *via* parenteral routes,^4,5^ and delivery to particular sites *e.g.* lung.^6,7^

Polymer-based DDS, and in particular those based on amphiphilic block copolymers that consist of a hydrophilic block and a hydrophobic block and can self-assemble when in aqueous medium,^8–10^ have attracted a lot of attention. One example of such amphiphilic polymers are the monomethoxy poly(ethylene glycol)-*b*-polycaprolactone (mPEG-*b*-PCL) diblock copolymers.^11–17^ Both mPEG and PCL are polymers that are well-tolerated by humans and approved by the Food and Drug Administration (FDA) for DDS. PEG is amphiphilic, soluble in organic solvents, has a good toxicity profile, is industrially available at a low cost^18^ and has been used extensively in DDS. PCL is a biocompatible, hydrophobic polymer of semicrystalline nature. The presence of the ester groups in the polymer backbone makes it biodegradable as they can be cleaved usually by passive hydrolysis or potentially by enzymatic reaction.^19^ The degradation behaviour can also be controlled *via* the length of the PCL block.^20^ Co-polymers based on mPEG-*b*-PCL can be synthesised *via* ring opening polymerisation (ROP); the mPEG is the macroinitiator and the catalysts are Lewis acid metal centres such as stannous octanoate,^11,21^ or calcium ammoniate^12^ or organic catalysts including 1,5,7-triazabicyclo[4.4.0]dec-5-ene (TBD).^22^ Consequently, mPEG-*b*-PCL polymers have already been investigated for the delivery and controlled release of many drugs, including anti-cancer drugs^23,24^ and, of particular interest to us here, the nonsteroidal anti-inflammatory drug (NSAID) indomethacin.^16,25,26^

Indomethacin has low aqueous solubility (0.937 mg L^−1^ at 25 °C ^27^), a property shared by an estimated 40% of existing active pharmaceutical ingredients (APIs) and 70–90% of the new candidate APIs.^28,29^ This low water solubility leads to poor bioavailability^30^ and can result in uncontrollable precipitation after dosing.^31^ In order to improve formulation performance and to achieve a high surface area to volume ratio, particle size reduction, either *via* top-down or bottom-up methods, has been established in the pharmaceutical industry as a common formulation strategy.^32,33^ However, production of pure drug nanoparticles can be difficult to achieve and usually results in the rapid aggregation of the nanoparticles back into larger particles unless the particles are stabilised, typically by surfactants, or *via* other interactions.^34^

An alternative strategy is to incorporate the API into more complex nanoparticulates which can subsequently release the drug. One method to produce polymer-based drug bearing NPs is *via* nanoprecipitation,^33,35,36^ also known as “co-solvent” or interfacial deposition method.^9^ In nanoprecipitation, two solvent phases are required; an organic phase where the amphiphilic polymers and the API are dissolved and an anti-solvent medium where the polymers are insoluble (in most cases this means water). The organic phase is added in the anti-solvent, either by a drop method^37,38^ or by pumping the two solvent streams in a mixer (flash nanoprecipitation FNP^33,39,40^). Both methods lead to the formation of the polymeric NPs due to polymer chain collapse *via* hydrophobic chain–chain association. During this process, drug molecules in the vicinity can be encapsulated into the polymer matrix, forming the final polymer–drug NPs. mPEG-*b*-PCL indomethacin loaded NPs have been produced in the past *via* a drop-nanoprecipitation method with the highest drug loadings reported ranging between 16–42%.^10,16,25,41^

Tuning the parameters to maximise the efficiency of such NP preparation methods is not trivial. In order to investigate factors like polymer lengths, solvent selection, mixing times, mixer geometries, as well as more fundamental issues like polymer–drug interactions, a variety of computational methods such as computational fluid dynamics (CFD) and molecular dynamics (MD)^42–46^ have previously been applied. MD in particular has been used to calculate solubility parameters,^47–49^ polymer–drug encapsulation^46,50^ and polymer self-assembly.^43,51–54^ Through such studies, MD has provided invaluable insight into the interactions that take place at an atomic level, and so link fundamental principles of physics and chemistry to the emergent behaviour of these complex systems. To date however, nanoprecipitation in particular has been modelled most usually using lower resolution computational methods, due to the large size of the systems to be simulated, and long timescales required; dissipative particle dynamics^34,55^ is a popular approach. Recently though we have demonstrated the application of a multiscale modelling approach, where the solutes can be studied in atomistic detail while the solvent is treated in a low-resolution manner.^46^ These studies have proved very insightful, however a detailed all-atom simulation of nanoprecipitation where all interacting species are modelled at full resolution is desirable, and so far has been missing from the field.

The objective of this work was to develop a method of encapsulating a drug nanoparticle in a thin polymer layer, to increase the drug loading while stabilising the drug nanoparticle. The objective also included some understanding of the mechanism of the encapsulation method through a parallel investigation using computational modelling. The present work is based on a similar concept developed in the polymer encapsulation of iron oxide nanoparticles using the interfacial deposition method.^56,57^ In this prior work,^57^ iron oxide nanoparticles with a thin layer of polymer surrounding the NP were generated and we therefore adapted this method for the preparation of hydrophobic drug-containing nanoparticles. We describe here, the initial stages of this method development to drug nanoparticles combining both top-down (macro to nano) and bottom-up (molecular to nano) approaches. The drug nanoparticles were formed from a top down method, *via* a simple sonication in water pre-step, while the polymer coating was applied by a bottom up method. Ultimately, we demonstrate here a method that provides excellent batch-to-batch reproducibility and results in nanoparticles with high drug loadings (up to 78%). At the same time, computational simulations of the polymer coating procedure using fully atomistic MD simulations, provides insights into how and why this nanoparticle production and coating process takes place.

## Materials and methods

### Molecular dynamics methodology

Details of all non-standard data files, input scripts, and simulation protocols is included in the ESI.†

### System preparation

**Fig. 1:**
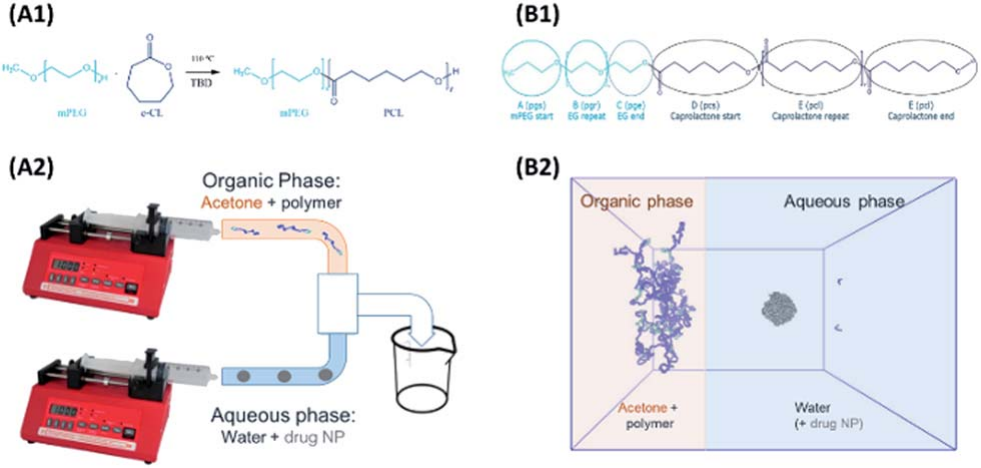
(A1) Ring opening polymerisation of ε-CL using monomethoxy poly(ethylene)glycol as the initiator and TBD as the organic catalyst. (A2) The interfacial deposition method using two syringe pumps, where the organic phase containing the polymer is mixing in a T mixer with the aqueous phase containing just water (polymeric NPs formation) or water with drug particles (polymer–drug NP formation). (B1) The polymer residues for the MD simulations (B2) the start of the MD polymer–drug NP formation simulation, replicating the mixing of the two phases as occurring in the T mixer.

Models and forcefield parameters for the polymers were developed as follows. First a “minimal” model for a mPEG-*b*-PCL polymer, consisting of one of each of the required start, repeat, and end units, was created using ChemDraw3D (Fig. 1, B1). This was then submitted to the online Automated Topology Builder (ATB) tool^58,59^ that provided appropriate parameters for use with the GROMOS53a6 or 54a7 (ref. 60 and 61) force-fields. The model was then dissected into individual monomer components (residue topology files) that could then be reassembled into polymers of any desired composition/ratio (using the *tleap* tool of AMBERtools^62^). Parameters for indomethacin were also obtained *via* the ATB service. All topologies and parameters for the models used in this work can be found in the ESI.†

The interfacial deposition method was envisioned as a biphasic model, where two regions, the organic and the aqueous, were allowed to mix (Fig. 1, B2). A large cubic box (edge of 26.7 nm) was built using Packmol^63^ that contained the aqueous region. For the coating studies a 5 nm (diameter) amorphous indomethacin sphere, containing 145 molecules, was placed in the centre of the box. The box was solvated in GROMACS using the *gmx solvate* tool, using the SPC water model,^64^ resulting in a 4.12 mg ml^−1^ indomethacin in water suspension. The box was then expanded in the *x* direction in such a way to accommodate the acetone–polymer phase, addressing two critical parameters: (a) the acetone : water molar fraction (*x*_a_ = 0.089), corresponding to the experimental setup where 2 ml of acetone are mixed with 5 ml of water and (b) the number of the polymer chains needed hypothetically to coat all of the particle' surface, but still at a reasonable polymer : acetone concentration.

### Running parameters

The GROMOS53a6 forcefield was used for the earlier studies of polymer equilibration, acetone–water miscibility and initial biphasic test-studies. With the release of the newest 54a7 forcefield, the latter was selected for all biphasic simulations.^60^ The cut-off distance for the van der Waals and the electrostatic interactions was set to 1 nm. All MD simulations were run in GROMACS (versions 4.6.5 and later 5.1.0)^65^ using either resources of the high performance computing (HPC) cluster of the University of Nottingham or on the UK National Supercomputer ARCHER.^66^ Visualisation of the trajectories and visualisations used in this work were performed using VMD.^67^ After the solvation of the biphasic box in GROMACS using the SPC water model,^64^ the atoms were relaxed by 10 000 steps of EM with the steepest descent algorithm. Then each of the two phases were equilibrated separately, *via* NVT ensembles at 298 K (V-rescale thermostat) for 100 ps and then in an NPT ensemble at 1 bar (Berendsen barostat) was applied to the whole system for another 100 ps. Bonds to hydrogen atoms were constrained to allow a time step of 2 fs. All of the MD simulations were run with the same barostat and thermostat, under PBC. Long-range electrostatics were treated with PME^68,69^ At the end of each equilibration step (EM, NVT, NPT) of the previous processes, the potential energies, temperature and pressure were checked retrospectively to ensure the proper equilibration of the system.

Analysis of the simulations was achieved using the built-in GROMACS tools; *gmx distance* and *gmx mindist* modules were used to calculate the distances between the centre of masses, and the number of contacts, retrospectively, of two groups of interest. *gmx density* was used to calculate the distribution of the solvent molecules across a box direction in order to evaluate whether the two solvents have adequately mixed within the simulation time. The *gmx msd* module was used to calculate the diffusion coefficient *D* of acetone in water. Solvent evaporation was replicated computationally as follows: from the final configuration of the system, a percentage of the acetone molecule were randomly chosen, removed, and replaced with water molecules (4 waters per acetone molecule). After energy minimization 10 ns of MD was run. The process was repeated five times, removing 20%, 30%, 40%, 50%, and 50% of the remaining acetone molecules respectively each cycle.

### Experimental materials and methodologies

All chemicals were bought from Sigma Aldrich unless stated otherwise. Monomethoxy poly(ethylene glycol) (mPEG) with molecular weights 350, 550, 750, 2000 g mol^−1^, ε-caprolactone (ε-CL) and the catalyst 1,5,7-triazabicyclo[4.4.0]dec-5-ene (TBD) were used as received and were kept under nitrogen atmosphere. Dichloromethane (DCM) and chloroform (CHCl_3_) HPLC grade were purchased from Fischer Scientific. Indomethacin (Lot #BCBK0293V) was purchased from Sigma Aldrich and was used as received.

### Polymer synthesis

mPEG-*b*-PCL copolymers were synthesised *via* ROP using the mPEG chains of different molecular weight as the initiators (Fig. 1A)^70^ and the organic catalyst 1,5,7-triazabicyclo[4.4.0]dec-5-ene (TBD).^22,71,72^ All items of glass equipment (syringes and round bottom flasks) were kept in an oven overnight at 150 °C to remove any residual moisture. Prior to the reaction they were placed under a fume hood and a magnetic stirrer was added to the round bottom flask which was quickly capped with a rubber septum. A constant nitrogen flow was introduced to prevent the presence of moisture, and the flask was heated to 110 °C. The mPEG was added to the flask, (either with a syringe or prior to capping) and the system was left under nitrogen for an extra 20 minutes before the addition of the ε-CL. A fixed molar ratio of [mPEG] : [ε-CL] [1] : [40] was maintained in all the reactions. The ε-CL was added drop-wise *via* a glass syringe and a sample was collected for the determination of the initial monomer units *via*^1^H NMR. The reaction started with the addition of the catalyst (TBD dissolved in DCM, 2% mol mol^−1^ with respect to ε-CL). Maximum conversion to polymer was achieved after 30 minutes when the reaction was terminated with the exposure of the system to air. The result was a very viscous liquid that after cooling to room temperature needed to be dissolved in chloroform for the following purification step. The polymers were purified *via* precipitation in cold methanol for the removal of any unreacted monomer and the catalyst.

### Polymer characterisation

^1^H-NMR spectroscopy was used to determine the degree of polymerisation and the purity of the final polymers. All NMR samples were prepared with CDCl_3_, run in a Bruker DPX UltraShield spectrometer (400.1 MHz) at 25 °C and were analysed with the MestReNova software (version 8.0.2) of MestReLab. GPC was carried out using a PL50+ Polymer Laboratories system, employing two mixed bed (D) columns at 30 °C, using chloroform (CHCl_3_) as the mobile phase, flow rate 1 ml min^−1^ equipped with a refractive index detector. Polystyrene standards (*M*_n_ range: 443 000 to 132 g mol^−1^) were used to calibrate the GPC. Molecular weights and polydispersity (*Đ*) values were calculated using Polymer Labs Cirrus 3.0. Water Contact Angle (WCA) measurements were conducted at 25 °C using a KSV Cam 200 (KSV Instruments Ltd., Helsinki, Finland) equipped with a dedicated software (CAM200). Samples were prepared by coating glass microscope slides with polymer thin films *via* solvent evaporation from 5% w/v solutions of polymers in acetone. Attenuated total reflectance infrared spectroscopy (ATR-IR) spectra were recorded with an Agilent Technologies Cary 630 FTIR equipped with a diamond single reflection ATR unit. Spectra were acquired with a resolution of 4 cm^−1^, in the range 4000–650 cm^−1^ by recording 32 interferograms. Differential scanning calorimetry (DSC) (Q2000, TA Instruments, Leatherhead, UK) experiments were conducted at a heating rate of 10 °C min^−1^. Thermal Analysis Software (Version 4.5.05A) was used for data analysis.

### Preparation of nanoparticles

Unloaded polymer nanoparticles were prepared following an interfacial deposition method^9^ previously adapted for the preparation of polymer coated iron oxide nanoparticles (IONPs).^57^ The method is based on solvent displacement and is schematically described in Fig. 1B. The organic phase was prepared by adding various amount of polymer (0.1, 0.2, 1, 0.2, 10 and 20 mg) in acetone (2 ml). The acetone–polymer solution was loaded in a glass syringe and used in the organic phase pump. In the aqueous phase, freshly filtered HPLC grade water (5 ml) was used. The volumes of the two phases were kept constant in all the experiments. The two pumps started the mixing of the two phases simultaneously (organic phase flow rate 0.84 ml min^−1^, aqueous phase flow rate 2.4 ml min^−1^). The solvents were introduced to the connecting tubes (PEEK, organic solvent resistant, 0.25 mm internal diameter bought from Kinesis UK) and were mixed using a T-connector (Kinesis). The mixture was then collected in a vial containing a magnetic stirrer. The resulting solution was stirred overnight for the evaporation of acetone and was collected for further analysis.

The drug-bearing aqueous phase was prepared by adding solid indomethacin into a round bottomed flask containing 50 ml water (HPLC grade, filtered) in order to achieve different theoretical concentrations (1, 0.5, 0.25, 0.1 mg ml^−1^). The flask was then sonicated for 15 minutes resulting in a milky suspension which was immediately used in the coating experiments.

Polymer coated indomethacin nanoparticles were prepared with the same procedure as the polymer-only nanoparticles; the organic phase of the acetone–polymer solution (2 ml) and the drug-bearing aqueous suspension of the freshly sonicated indomethacin (5 ml) were mixed using the syringe pumps (organic phase flow rate 0.84 ml min^−1^, drug-bearing aqueous phase flow rate 2.4 ml min^−1^). After the evaporation of acetone overnight, the resulting nano-suspension was collected and an extra purification step was introduced to ensure the removal of any uncoated drug particles; the suspension was centrifuged (3 minutes at 2000 rpm) to sediment the uncoated free drug, which tended to aggregate. The supernatant containing the polymer coated drug nanoparticles was collected and was used either as collected for the size measurements or was lyophilised for the determination of the drug loading.

### Nanoparticle characterisation

Nanoparticle size was determined by dynamic light scattering (DLS) at 25 °C, using a Malvern ZetaSizer Nano ZS at a scattering angle of 173° and laser of 633 nm. All samples were measured in triplicate for the report of the average values (intensity distributions).

The critical aggregation concentration above which polymer chains will collapse to form nanoparticle aggregates was measured by DLS; a suspension of polymer NPs (1 mg ml^−1^) was diluted in water in a concentration range (300–0.1 ml mL^−1^). Using the highest concentration (300 mg ml^−1^) the attenuator of a Malvern ZetaSizer Nano ZS was selected and kept constant during the rest of the measurements. The count rate (kcps) was recorded for each of the decreasing polymer concentrations. The concentrations were plotted against the count rates.

The colloidal stability of the NP formulations was investigated using increasing salt concentrations. Barium chloride (BaCl_2_) in a range of concentrations (0.16–0.66 M) was added to 0.5 ml of the NP suspension and was left for 10 minutes. If a precipitation of the NPs was observed, then this was considered to be the maximum salt concentration at which the NPs were stable.

Zeta potential measurements were conducted at 25 °C, using a Malvern ZetaSizer Nano ZS. The nanoparticle suspensions were used without any further treatment and were run in triplicate for the report of the average values.

Transmission electron microscopy (TEM) was used to characterise the size and morphology of the nanoparticles. The sample in aqueous suspension (13 ml) was added to a copper grid (formvar/carbon film 200 mesh copper (100)) was left on the grid for 10 minutes and then the excess was removed *via* a filter paper. Then, freshly prepared uranyl acetate (2%, 13 ml) was added on the grid and was left for 5 minutes before the removal of the excess with a filter paper. The grid was allowed to dry under a fume hood for a minimum of 30 minutes prior to use.

Polarised optical microscopy (POM) was used to validate the physical state of the NPs. An Advanced Polarising Microscope (Prior LuxPOL™ with 12 V and 30 W halogen lamp) was used. The nanosuspensions were added *via* a pipette on a glass slide and pictures were collected with and without the polariser.

Quantification of drug loading was achieved *via* UV-Vis spectroscopy using an Agilent UV-Vis spectrometer. The calibration curve for indomethacin was prepared in an acetone : methanol mixture (80 : 20) measuring the absorbance at 350 nm. The lyophilised samples were dissolved in the solvent mixture and their absorbance was measured. Drug loading and entrapment efficiency were calculated as follows:1
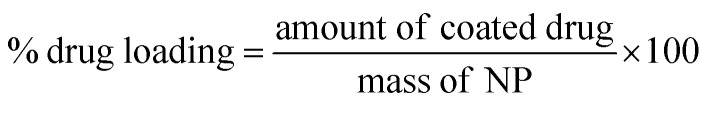
2
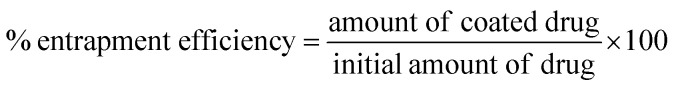


To account for the drug losses that were observed both during the syringe loading with the indomethacin suspension and during the experiments (discussed in detail in the Results section), the latter eqn (2) was modified to the following:3
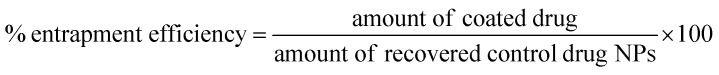


## Results and discussion

The nanoprecipitation methodology in the presence of nanoparticles can produce either polymer coated nanoparticles, or a mixture of polymer nanoparticles and drug nanoparticles, so control experiments were required in both computational and experimental work to determine which particles are present and the relative size ranges of the different alternative products. These were also important in determining whether there is agreement between the computational and experimental results.

### Molecular dynamics simulations

The purpose of our molecular dynamics simulations was to provide atomic-level insights into the NP formation process. This was technically challenging due to the complexity and novelty of the system, from a computational perspective. Firstly, as the whole NP formation process is driven by the acetone–water diffusion, selecting the correct acetone model was required. Despite the existence of the various acetone models,^73–78^ modelling acetone–water diffusion has proven to be challenging and the modelling community still lacks an adequate acetone model to use in the GROMOS53a6 & 54a7 forcefields.^43,61,76,79,80^ After testing the existing models in trial systems and following past work from our group,^46^ it was decided to use the ATB-derived acetone model, which demonstrated good miscibility with water (topologies at ESI†).

In order to validate the behaviour of the molecular components in this work, it was necessary to perform a series of control simulations of the polymers and the drug NP in the presence of pure solvents. In simulations, the mPEG_350_PCL was soluble in acetone (ESI Fig. 1†) but formed aggregates in water (ESI Fig. 2†). The same was observed when an indomethacin NP of 5 nm diameter was used (ESI Fig. 3 and 4†), thus demonstrating that the basic physicochemical properties of the polymer and drug components reflected experimental conditions.

Experimentally, solvent displacement methods are used widely to produce drug loaded NPs.^20,81,82^ In such systems, the polymer and the drug are dissolved in an organic solvent, which is then mixed with water. The mixing of the solvents is performed either *via* the drop-by-drop addition of the organic phase into the water (batch method), or by using mixing of streams of the two fluids (flow method). In both cases, the solvent displacement causes the hydrophobic part of the polymers to aggregate and form NPs. Typically, in these systems a large excess of polymer is used together with dissolved drug, usually resulting in low yields and encapsulation efficiencies. The system presented in this work differs from the above in regard to the drug-bearing phase (which is in nanoparticulate form) and in the relative amount of polymer (which is much lower relative to the amount of drug). Indomethacin has a very low aqueous solubility^27^ so the relative solubility of the drug in both phases is important to the outcome and the MD system was designed as a biphasic one (Fig. 1C). Every attempt was made to match realistic experimental conditions; however, a compromise must be made to ensure that the system is large enough to be representative, but at the same time small enough for the simulation to be completed at a reasonable computational expense. Due to the latter, only the simulations using mPEG_350_PCL were carried out.

**Fig. 2:**
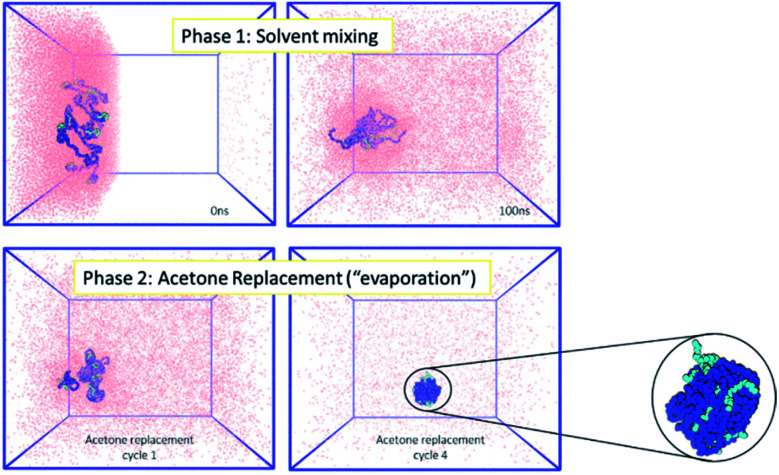
(Left) MD simulations for the formation of polymeric NPs, where the polymer chains (mPEG: light blue, PCL: dark blue) are in the organic phase (acetone: pink). Acetone mixes with water (not shown for clarity) during phase 1 and is then removed during phase 2 to mimic the evaporation that occurs in the experimental environment. (Right) At the end of the simulation a single polymeric NP of 3.4 nm diameter has been formed.

Firstly, the behaviour of the polymers in this biphasic system was investigated. As expected from the experimental results, during simulations the PCL chains start to aggregate, as the polymers are exposed to more water molecules as they diffuse in from the aqueous compartment. Due to the continuing presence of the acetone in the system, the polymer NP has not fully formed by the end of 100 ns simulation. However, during the following acetone evaporation simulations, the hydrophobic PCL blocks of the polymer chains aggregate more firmly, leading to the formation of a dense polymer NP of 3.4 nm diameter (Fig. 2 and ESI Fig. 5, 6†).

The next control investigated the fate of an indomethacin drug NP in a solvent mixing simulation in the absence of the polymer. In an attempt to replicate the expected experimental aqueous phase where indomethacin nanoparticles would be in a suspension, a 5 nm diameter amorphous indomethacin NP was placed in the aqueous region of the biphasic box while the acetone region was polymer-free. The simulation ran for a total of 100 ns. The drug NP stayed intact until 70 ns while after that it started to slowly swell up and dissolve, due to the increasing concentration of acetone around it (ESI Fig. 7†).

**Fig. 3:**
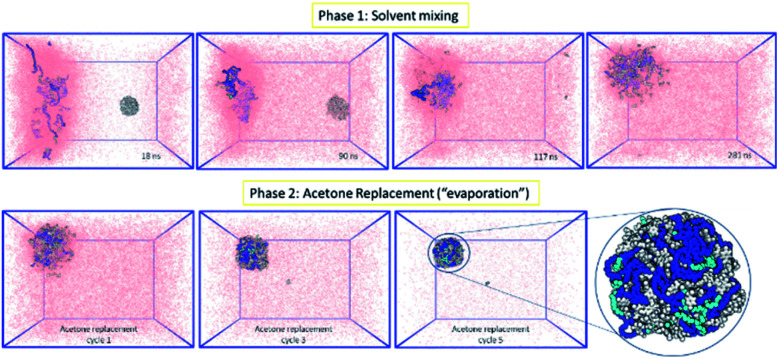
MD simulation for the formation of a polymer-coated indomethacin NP (colour scheme as in Fig. 2) (Top) phase 1 for the simulation where the two regions, acetone and polymer on the left and water and indomethacin on the right side, are allowed to mix freely. As the indomethacin NP reaches in close proximity with the polymer chains it is dissolved. The indomethacin molecules are then interacting freely with the polymer chains for 150 ns. (Bottom) Phase 2 of the simulation process where every 10 ns acetone molecules are replaced with water molecules, mimicking acetone evaporation and thus facilitating the self-assembly of the polymer–drug NP. The final NP is seen as dissolved indomethacin molecules in a polymer matrix.

Following the rationale of the previous simulations, the complete system comprised of both the indomethacin aqueous suspension phase (a cubic solvated box with the 5 nm diameter amorphous indomethacin NP in the centre) and the polymer-rich organic phase (31 mPEG_350_-*b*-PCL_2000_ polymer chains dispersed in acetone). The simulation box contained more than 2.6 million atoms (Fig. 3). The simulation ran for 281 ns (phase 1, ESI Fig. 8†), followed by 5 simulation sets of 10 ns each, to mimic the evaporation of the acetone (phase 2, ESI Fig. 9†), resulting in a total simulation time of 330 ns. As the acetone and water molecules began to mix, the polymer chains in the acetone region started to aggregate, while the drug NP diffused around the water region but stayed intact. However, at around 100 ns the drug NP encountered the nascently aggregating polymer and dissolved in it. In order to identify the exact time point where the interactions between the polymer chains and the indomethacin NP occurred, the number of contacts, the distance between the PCL blocks and the indomethacin molecules as well as the radius of gyration and end-to-end distances of the polymer chains were calculated (ESI Fig. 10 and 11†). The complete dissolution of the indomethacin NP was not observed during the control simulation (acetone, no polymer) and thus suggests that there is a synergistic effect between the polymer and the acetone that breaks down the drug NP. At the same time, as both the indomethacin NP and the polymer chains are soluble in acetone, and the acetone is still present, the formation of a dense polymer–drug NP is inhibited. Once the acetone molecules started to be replaced by water molecules the polymers and drug particles started to interact strongly and a particle started to form; a polymer–drug NP of 7 nm in diameter was obtained. This resulting NP, after nearly 330 ns of simulation, did not resemble the ideal coated NP as envisioned in the Introduction of this paper. The mPEG chains were entangled with the drug molecules and since the drug NP had dissolved completely during the first 110 ns of the simulation, the drug molecules were dispersed uniformly inside the NP. The polymer chains incorporated 144 indomethacin molecules resulting in a 99.3% yield with respect to the drug. Only one indomethacin molecule (∼0.69%) remained unattached to the polymer–drug NP and was moving freely in water.

## Experimental

### Polymer characterisation

The polymers were synthesised as described in the methods section employing a simple solvent-free ROP synthetic strategy. The chain propagation and formation of mPEG-*b*-PCL diblock copolymers was studied *via*^1^H NMR. The proton signal at *d* 4.2–4.27 ppm corresponding to the CH_2_ methylene protons adjacent to the ester bond to the PCL component was used for the calculation of the degree of polymerization (DP). The molecular weight of the polymer was calculated by the integration of peak c after setting peak b to the unit of 1, *i.e.* following the equation:

4 *M*_n_(NMR) = *M*_n_(mPEG) + 114.14 × DP

The two triplets in the region 3.2–3.3 ppm correspond to the catalyst TBD and were absent after the purification by precipitation in cold methanol (ESI Fig. 12†). The properties of the polymers after their characterisation *via* NMR and GPC are summarised in Table 1 (ESI Fig. 13–16, ATR-IR in ESI Fig. 17†). The hydrophobic segment of the polymers, experimentally analysed, was very close to the targeted value of 2000 Da and for this reason the polymers will be referred simply according to the length of their mPEG chain. Further characterisation by DSC (ESI Fig. 18–21† and contact angle (ESI Fig. 22†)) showed that the increase of the hydrophilic mPEG block length resulted in a decrease in the contact angle and a decrease of the PCL's crystallinity (ESI Fig. 23†). Melting points of the polymers were in the range of 50–59 °C and no glass transition was detected for any of the block copolymers, probably because these were likely to fall below the minimum temperature of detection by the adopted instrument^83^ (−40 °C).

**Table 1:** Molecular characteristics of the mPEG-*b*-PCL copolymers

Sample	NMR		GPC		DSC	
	*M*_n_ PCL^a^	*M*_n_ polymer^b^	*M*_n_	*Đ* c	*T*_m_ (°C)	Δ*H* (J g^−1^)
mPEG_350_–PCL_2000_	1940	2290	7380	1.6	50.2	78.7
mPEG_550_–PCL_2000_	2060	2610	6820	1.6	55.7	82.3
mPEG_750_–PCL_2000_	1940	2690	7600	1.4	50.1	74
mPEG_2000_–PCL_2000_	2170	4170	10 960	1.5	59.1	82.5
mPEG_350_			390	1.2	−6.8	49.1
mPEG_550_			680	1.1	14.4	129.6
mPEG_750_			980	1.1	29.7	145.4
mPEG_2000_			3300	1.1	51.9	171.7
ε-CL					−1.3	119.2

a Calculated by integration of peak b of the ^1^H NMR spectrum.

b Calculated by *M*_n_(mPEG) + *M*_n_(PCL).

c Polydispersity (*M*_w_/*M*_n_).

### Formation and characterisation of polymer nanoparticles

In order to distinguish the polymer-coated drug NPs from polymer NPs, the formation of polymer micelles was investigated for all the polymers.

**Fig. 4:**
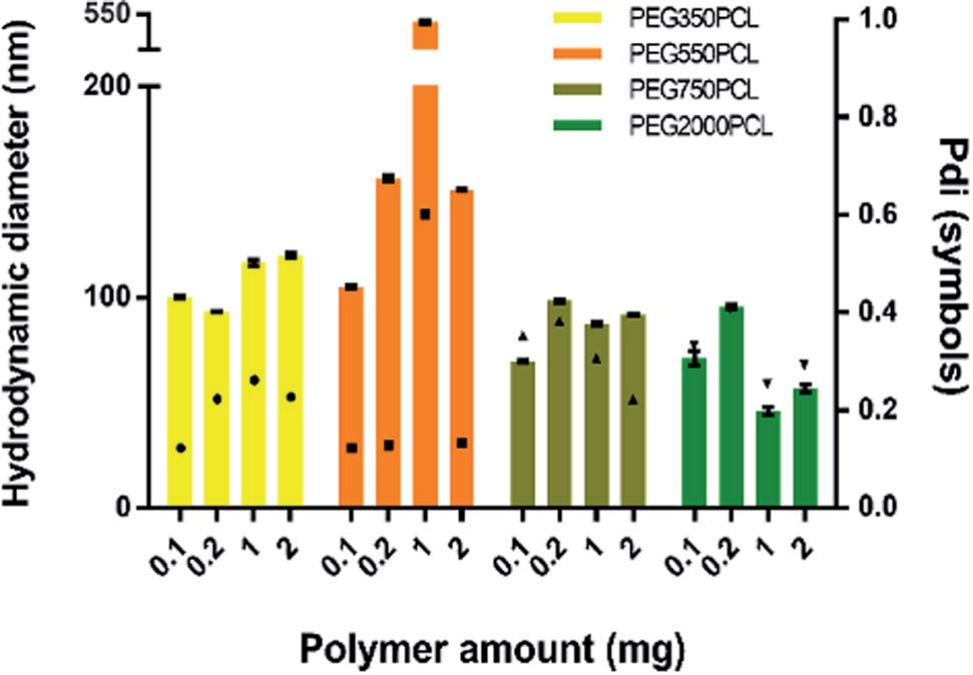
Left *Y*-axis: size (intensity) of the polymeric nanoparticles using various polymer amounts (bars) for each different polymer. Error bars represent the standard deviation of three measurements on a single batch. Right *Y*-axis: PDI of measurements (symbols).

Large particles that sedimented within 24 hours were formed for the mPEG_550_PCL and mPEG_750_PCL polymers in the range 2–1 mg (Fig. 4); this led to the decision to use polymer amounts in the range of 2–1 mg for the rest of the polymers. In Fig. 4, the *z*-average size (intensity distribution) and polydispersity data are presented (bars and points respectively) for a range of different polymer nanoparticle preparations from the four different polymers using a range of different amounts of polymer. Particles formed from mPEG_350_PCL demonstrated low polydispersities, indicating that a well-defined NP population was present. mPEG_550_PCL formed the largest NPs with diameters more than 150 nm (with the exception of 0.1 mg where a 100 nm *z*-average size was recorded). It also showed single peaks of NPs with PDIs less than 0.2 with the exception of the NPs formed at 1 mg polymer, which precipitated upon the removal of acetone. Polymer mPEG_750_PCL formed NPs of less than 100 nm in diameter but the increased PDI values (between 0.30–0.38) in the lower concentrations suggested sub-optimal stability. As expected, due to the increased mPEG length and thus its steric stabilisation properties, mPEG_2000_PCL demonstrated the smallest range of NP size with the differing polymer amounts, showing sizes ranging from 80 nm (0.2 mg) down to 50 nm (1.0 mg) in diameter. The reproducibility of the method was demonstrated by preparation of 4 batches of the mPEG_350_PCL at 0.1 mg of polymer which successfully replicated the same size distribution *via* DLS (ESI Fig. 24†).

Zeta potential measurements were performed to evaluate the surface potential of the polymer NPs (ESI Fig. 25†). All the polymer NPs with the exception of mPEG_750_PCL (−16.1 mV) demonstrated zeta potential values lower than −20 mV. Zeta potential values less than |30| mV suggest borderline unstable behaviour^84^ for NP populations whose stability depends on electrostatic forces. However, the polymer NPs produced in this work were expected to be sterically stabilised to varying extents relating to the length of the hydrophilic mPEG block.

In order to evaluate the NP stability further, a salt concentration test was performed using barium chloride (BaCl_2_); the increase in the ionic strength of the solution destabilises both electrostatic and sterically stabilised particles, causing the precipitation of unstable populations. For the NPs produced with the lowest polymer amount (0.1 mg), the polymer NPs started precipitating when the salt molarity reached 0.66 M (as judged visually), indicating a stable population of NPs. The physical stabilities of the NPs were also assessed. When kept under typical lab conditions (stored in a cupboard at room temperature), the NPs exhibited the same particle distribution after 1 month (ESI Fig. 26†). TEM was also used to characterise the polymeric NPs. (ESI Fig. 27†).

### Preparation of the indomethacin-bearing aqueous phase

Initial experiments evaluated both nanomilling and cryomilling as possible methodologies for producing nanoparticle drug suspensions, but these were less convenient and provided no advantages compared to a simple sonication of crystalline indomethacin (DSC at ESI Fig. 28†) in water for the preparation of an aqueous suspension of drug. DLS measurements just before the start of the coating process revealed a population of sub-micron particles with an average diameter of 712 (±357) nm and a high polydispersity, indicative of the instability of the suspension, which had a tendency to form large aggregates at the surface of the water and precipitate in a timeframe of 2 hours (ESI Fig. 29†). Further analysis of the suspension by TEM (Fig. 6A) and polarising optical microscopy (ESI Fig. 30†) showed large aggregates with clear, sharp crystal planes. These suspensions were used in the interfacial deposition as the aqueous drug-rich phase.

### Formation and characterisation of polymer-coated drug nanoparticles

**Fig. 5:**
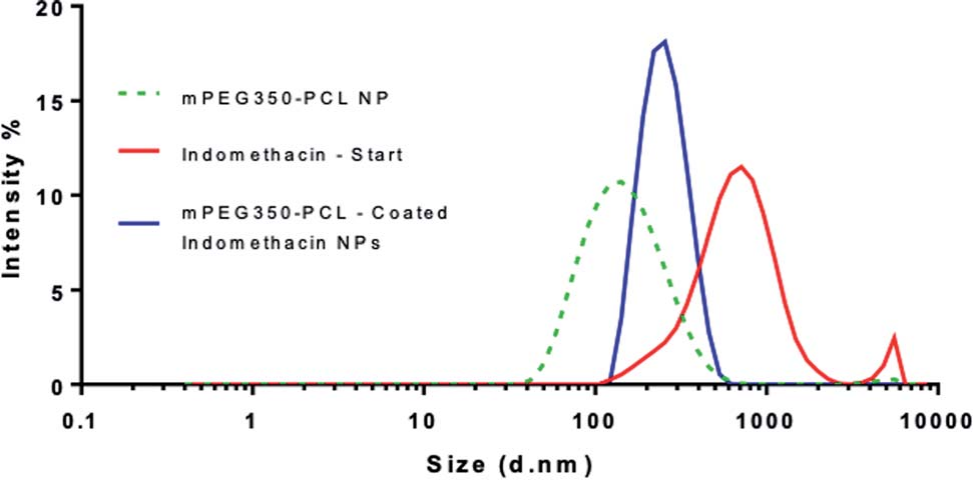
Overlay of 3 separate DLS measurements of nanoparticles produced using the highest amount of polymer (2 mg). The peak corresponding to the polymer-coated drug NPs (blue) is shown in comparison with those of the starting indomethacin population (red) and the polymeric NP (green – dashed). This analysis was carried out using polymer coated drug nanoparticles which had been centrifuged to remove aggregated uncoated drug NP.

When the coating method was performed with mPEG_350_PCL (0.1 mg) in the presence of the white, opaque drug suspension (1 mg ml^−1^), fine translucent nanosuspensions were formed (ESI Fig. 31 and 32†) that had different size distributions when compared to their drug-free polymer NP equivalents. The same behaviour was observed for all the different polymer amounts selected and for all the polymers as well. With time, some larger particles appeared due to a small amount of uncoated drug nanoparticles which aggregated. These aggregates could be easily removed using a short centrifugation which resulted in the improvement of the PDI of the DLS measurements (ESI Fig. 33†) and drug aggregates were not detectable by Polarised Optical Microscopy (ESI Fig. 34†). DLS analysis for the highest polymer amount (2 mg) is shown in Fig. 5 where the nearly micrometre sized population of drug particles (red) has disappeared and a single NP peak of polymer coated drug nanoparticles was obtained (diameter of 250 nm, blue peak in Fig. 5).

**Fig. 6:**
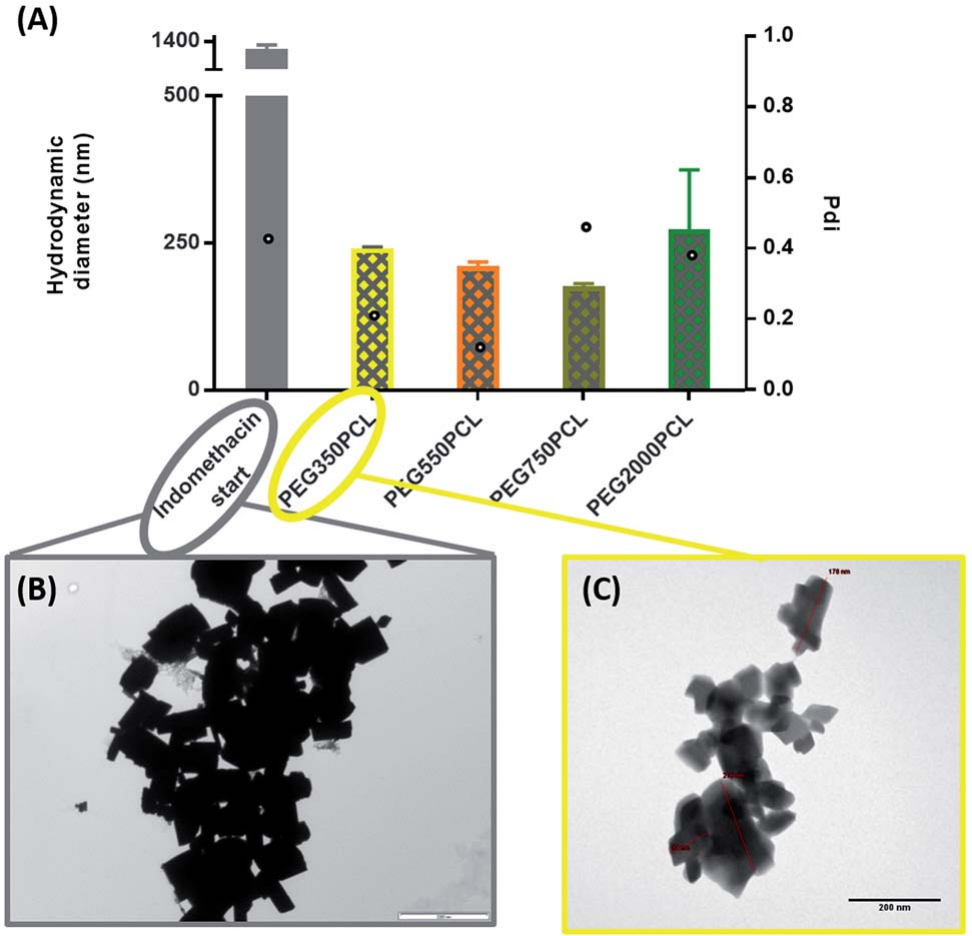
(A) DLS results of polymer–drug NPs for the 0.1 mg polymer amount. Bars represent the hydrodynamic diameter (left *y* axis) while circles represent the PDI (right *y*-axis). (B) TEM picture of indomethacin uncoated crystals. Scale bar 2000 nm. (C) TEM picture of mPEG_350_–PCL coated indomethacin crystals (lowest starting polymer concentration) scale bar 200 nm.

As illustrated in Fig. 6, all the polymers (0.1 mg polymer) resulted in the formation of polymer-coated indomethacin NPs with sizes in the range of 150–260 nm. However larger PDIs for the mPEG_750_PCL and mPEG_2000_PCL led to focusing the future experimental work on mPEG_350_PCL and mPEG_550_PCL coated drug NPs. TEM was employed to visualise the polymer-coated drug NPs (Fig. 6C) where it is clear that the coated NPs differ significantly from the starting indomethacin suspension (Fig. 6B) both in size and in morphology as they are smaller and do not exhibit the same sharp crystal edges. Although they appear as if they have aggregated, this may be at least partially due to the TEM sample preparation method.^71,85^ When larger amounts of polymer were used (mPEG_350_PCL 2 mg) the surrounding polymer coating was significantly thicker (ESI Fig. 35†). Preparation of 5 batches of PEG_350_PCL-coated indomethacin NPs resulted in the same size distribution by DLS (ESI Fig. 36†) demonstrating the reproducibility of the procedure. The suspensions were stable kept under typical lab conditions for at least 10 days (ESI Fig. 37†).

Different starting concentrations of indomethacin (0.1, 0.25, 0.5 and 1 mg ml^−1^ in water) were also investigated (ESI Fig. 38†); although no apparent trend was observed, decrease of the drug amount resulted in increase of the populations' PDI. Drug loading and yield of the method using UV-Vis showed that drug loading reached up to 78% for mPEG_350_PCL at 0.1 mg (ESI Table 1†), which is very high compared to the traditional batch preparation methods for matrix nanoparticles (<25% (ref. 86)). Similar results were found for the other polymers as well when 0.1 mg was used as the polymer in acetone concentration (PEG_750_PCL 77.96%, PEG_2000_PCL 72.06%, data not shown). Drug entrapment efficiency was initially calculated by the conventional route of comparing the amount of encapsulated drug recovered compared to the initial amount of drug (eqn (2), ESI Table 1†). However, this gave exceptionally low drug encapsulation. Since it was clear that there was significant mechanical loss of indomethacin from the aqueous stream throughout the mixing apparatus, a control experiment was run without polymer present in the acetone stream, and the amount of drug recovered from the output determined. Consequently, the entrapment efficiency was recalculated using the amount of control drug recovered, instead of the starting amount of drug to give more representative values for the entrapment efficiency (eqn (3), ESI Table 1†).

## Discussion

There have been very many investigations into the formation of polymer–drug nanoparticles *via* ‘nanoprecipitation’ methods but rather fewer involving drug dispersions with amphiphilic polymer coatings.^33,36,87,88^ Accordingly, at the outset of this work there were a number of critical questions relating to the mechanisms by which polymer-coated drug nanoparticles might form. We thus decided to pursue a parallel course of experimental and *in silico* work in order to understand the processes and limitations of the polymer-coating method for the formation of stable, well-defined drug nanoparticles.

In a typical nanoprecipitation method, the solute is dissolved in the presence of a stabilising molecule (*i.e.* polymer) in a favourable solvent, which is then added into a miscible solvent that acts as a non-solvent for both the solute and the stabiliser.^33^ In the case of flash nanoprecipitation (FNP),^35^ this is done by mixing the two streams in a confined space providing supersaturation conditions in order for both the solute and the stabiliser to precipitate simultaneously. This method has been used to produce β-carotene–PEG–PCL,^89^ itraconazole–poloxamer 407,^82^ and other polymer NPs.^90^ The final NP population is dependent on the solvent miscibility and molar ratios, the nature and the length of the hydrophobic and hydrophilic blocks of the polymeric stabilisers, as well as the mixing times and flow ratios. However in this work, the FNP was adapted to a pre-existing coating method (interfacial deposition^9^) where, in the former case, iron oxide nanoparticles were present in the aqueous environment.^57^ In comparison with existing NP formulation techniques, the present method utilised both bottom-up and top-down characteristics. The drug underwent comminution by sonication while a flow nanoprecipitation method was used to coat the NPs with polymer.

### Molecular dynamics computational aspects

Acetone is generally considered to be a safe solvent option easily removed from formulations and at the same time it is miscible with water at all molar fractions. The selection of the solvent is a non-trivial issue; water-solvent miscibility affects the size of the polymer NPS formed *via* solvent displacement methods,^91^ but it also affects the API solubility in the binary systems.^92^ From the modelling perspective of this work, the absence of an accurate acetone model has proven to be a challenge, however, whilst acknowledging the limitations of the acetone model used, the physical representation of the system from the simulations is satisfactory; the simulations reproduced the expected experimental behaviour of the pure systems *i.e.* the dispersive behaviour of the mPEG-*b*-PCL polymers and the behaviour of indomethacin NP in pure solvents. The previously reported solvent clustering around the PCL chains that inhibits their full aggregation^43^ was also observed in the present simulations.

During the mixing of the solvents in the presence of both the polymers and the drug NP, the water insoluble indomethacin NP remained intact for the first 100 ns. However, when the drug NP diffused to be in close proximity to the polymer chains it was dissolved by them, individual indomethacin molecules diffusing freely amongst and interacting dynamically with, the polymer chains for more than 150 ns. The dissolution also appeared to be facilitated by the constant presence of acetone molecules around the polymers. These observations, many of which were unexpected, demonstrate the power of MD simulations as a complement to experimental studies of interfacial deposition, providing microscopic insights into macroscopic behaviours. From a NP preparation perspective, this is the first time an unbiased all-atom MD simulation has resulted in the self-assembly of a polymer–drug NP, something that reportedly was missing from the field.^93^ Past work on solvent displacement processes *via* computational fluid dynamics (CFD)^42,44,94,95^ have provided invaluable information on parameters like solvents' velocities, molar fractions, and mixer geometries, and are able to predict the final NP size based on population balance equations. However, such methods cannot describe the internal morphology of the NPs. The NPs formed in this work are not covalently bonded; the individual polymers and drug molecules are dynamic, and in this case, this seems to be a vital aspect of their properties and behaviour.

### Experimental aspects

At the outset of this work we were hoping that the thin polymer coatings obtained using iron oxide nanoparticles^57^ could be replicated on drug nanoparticles but had significant concerns that the solubility of drug in solvent or different physicochemical properties of the drug and polymer used could adversely affect the outcome. In practice, experimentally quite similar outcomes were in fact obtained in these two pieces of work.

Polymer nanoparticles of small size (mean 100 nm) were obtained in the flow solvent displacement production, however, there was quite a broad dispersity of sizes dependent on the amount of polymer used and PEG chain length of the polymers involved. This is the sort of size range expected due to the low amount of polymer used. When the solvent displacement process was used in the coating procedure, an unexpected observation was the production of a transparent suspension of coated nanoparticles, with a much smaller NP size for the polymer coated nanoparticles than the starting drug nanoparticles. From the microscopy results (both POM and TEM), it can be seen that the drug particles are aggregates of many smaller particles, however, the polymer coated nanoparticles have a coating mainly around individual nanoparticles rather than the whole aggregate. This suggests that under the conditions of the coating procedure, the drug nanoparticles disaggregate and are coated, individually. As the TEM pictures of the polymer coated drug NP aggregates show a larger size than the DLS results, this suggests that some re-aggregation of the polymer coated drug nanoparticles occurs, particularly in the preparation of the TEM samples. This explanation is also supported by the computational studies which show that the polymer dissolves the surface of the drug particle in forming the drug coated nanoparticle, which may help in the process of drug nanoparticle disaggregation. Overall the size of the coated drug nanoparticles is clearly distinguishable from populations of both polymer nanoparticles and uncoated drug nanoparticles.

Again, from the microscopy, the polymer appeared to be in an even distribution on the drug nanoparticles, with no obvious polymer nanoparticles present. However, the appearance of small aggregates with time suggested that some uncoated drug NP may be present, but once these drug aggregates were centrifuged away, the resulting polymer coating suspensions were stable with time. Experiments attempting to optimise the polymer coated drug nanoparticle size by changing the concentration of the drug present in the syringes did not show an obvious relationship between drug concentration and final nanoparticle size, and it is likely that a range of parameters influenced this outcome. As the amount of drug is decreased, the relative ratio of polymer to drug will increase, thus it would be expected that particle size would increase due to an increased polymer coating thickness as seen with the decrease of drug concentration from 1.0 to 0.5 mg ml^−1^. Alternatively it is possible that, an increase in the polymer to drug ratio might enhance the dissolution of the drug in the polymer leading to a greater breakdown/separation of the drug particles leading to a population of smaller drug particles. This mechanism may account for the decrease in particle size seen with the decrease in drug concentration from 0.5 to 0.1 mg ml^−1^. Further experiments and analysis would be needed to confirm these possibilities. The stability of the drug coated nanoparticles over a period of 10 days has been shown (ESI Fig. 37†), and this would be expected because of the colloidal stability of the polymers as demonstrated by the colloidal stability of the polymer nanoparticles to barium chloride solutions. The high melting temperature and low *T*_g_ of the PCL block would also be expected to result in physically stable nanoparticle coatings.

### Correlation of molecular dynamics and experimental aspects

The number of molecules that could be included in the simulation was limited by the computational cost, but we could approximately reproduce experimental concentrations of components and component ratios. As a result, though the absolute size of, for example, nanoparticles that spontaneously formed during the simulations could only be approximately 3% as large as those observed in experiments, the processes observed that lead to their formation could be realistic. In particular, the observation that in simulations the polymer does not so much coat the nascent nanoparticle, as form an amalgam with it, help us propose a mechanism for what is observed experimentally.

**Fig. 7:**
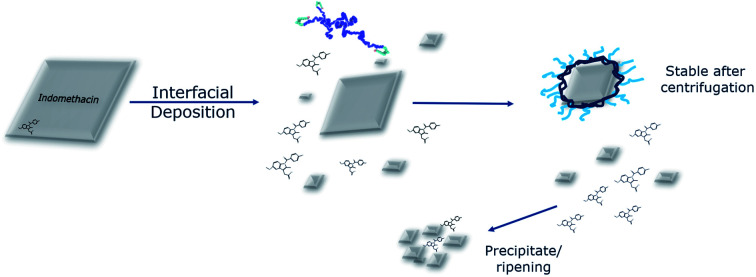
Interpretation of the phenomena that take place during the modified interfacial deposition. A population of micron-sized indomethacin particles in an aqueous environment is exposed to a flow of polymer and acetone. The drug particles are either broken down or dissolved due to the synergistic effect of the acetone and the polymers. The polymers are able to coat the surface of the drug NPs, making a population that can be stable due to steric interactions, while the rest of the uncoated drug particles aggregate and finally precipitate allowing removal by centrifugation.

In an attempt to interpret the interactions that take place based on the information collected from both the experimental and the computational studies, a graphical illustration is presented in Fig. 7. Micron-sized indomethacin particles are present in the aqueous phase. Once exposed under flow to acetone and polymers, their fate depends on their initial size: larger particles will have their surface dissolved, while smaller particles will dissolve completely to indomethacin molecules. This size reduction is not solely due to the presence of the acetone as the MD simulations have demonstrated that the polymers dissolve the indomethacin NPs as well. Also, in the light of the morphology of the polymer–drug NP as shown by MD, it is plausible to assume that with the larger drug nanoparticles used experimentally, the polymers could form a saturated surface, where polymer chains are entangled with dispersed indomethacin molecules, while the core of the drug NP remains crystalline.

It may be expected that some polymer micellisation may occur independently from the coating of the drug nanoparticles. The absence of a second size peak in the DLS measurements indicates that there was a preference for coating of the nanoparticle surface. This may depend on the existence of some polymer drug compatibility as has previously been suggested in polymer coating of iron oxide nanoparticles.

In the final nanosuspension, the nanosized indomethacin particles are polymer-coated and thus sterically stable. On the contrary, the uncoated indomethacin molecules or particles without the steric stabilisation will form larger aggregates that can be separated from the coated population by centrifugation.

We believe the presented work could aid the design and formulation of poorly soluble drugs. Polymer coated drug nanoparticles have an intrinsic advantage compared to matrix type nanoparticles as a result of the inherently higher drug loadings, even though these are dependent on how thin the polymer coated drug layer can be produced and/or how thick it needs to be to fulfil its delivery function. The very high drug loadings (over 75% w/w) already shown, lead to the possibilities for a much wider range of usages for drug nanoparticles in oral, parenteral or for other routes of delivery. The presence of a polymer coating on the drug nanoparticle has the advantage of providing colloidal stability compared to pure drug nanoparticles, and also that the surface coating then becomes dependent on the properties of the polymer rather than the drug. This method of polymer coating also has the advantage of not requiring high amounts of surfactant which can have several disadvantages in certain formulations. These advantages may give opportunities in common procedures for formulating nanoparticles, where previously the formulation of a number of different drugs depended on the surface crystal characteristics of the individual drugs. The methodology is very simple and reproducible from readily available materials and can be readily converted to a flow process method, making this methodology potentially attractive to pharmaceutical industry. All these characteristics add up to a wide range of possibilities across many different possible formulation areas.

## Conclusions

This work has combined both theoretical and practical approaches to investigate the formation of polymer-coated drug NPs. A modified solvent-displacement method was developed that produced mPEG-*b*-PCL coated indomethacin NPs with high drug loadings. In parallel, all-atom MD simulations of the nanoprecipitation method were performed, and provided a hypothesis to explain the experimental observations. We suggest that the combination of both aspects of the pharmaceutical field can provide a *nanoformulation-by-design* approach; using computational techniques in parallel with ‘wet-lab’ experimental work in order to understand and design better formulations.

## Conflicts of interest

There are no conflicts to declare.

## Supplementary Material

RA-010-D0RA00408A-s001

## References

[cit1] Chou L. Y. T., Ming K., Chan W. C. W. (2011). Chem. Soc. Rev..

[cit2] Jain K. K. , Methods in Molecular Biology, Humana Press, New York, NY, 2014, vol. 1141, pp. 1–56

[cit3] Cheng R., Meng F., Deng C., Klok H. A., Zhong Z. (2013). Biomaterials.

[cit4] Chiappetta D. A., Sosnik A. (2007). Eur. J. Pharm. Biopharm..

[cit5] Gaucher G., Dufresne M.-H., Sant V. P., Kang N., Maysinger D., Leroux J.-C. (2005). J. Controlled Release.

[cit6] Paranjpe M., Müller-Goymann C. C. (2014). Int. J. Mol. Sci..

[cit7] Yang W., Peters J. I., Williams R. O. (2008). Int. J. Pharm..

[cit8] Nishiyama N., Kataoka K. (2006). Pharmacol. Ther..

[cit9] Fessi H., Puisieux F., Devissaguet J. P., Ammoury N., Benita S. (1989). Int. J. Pharm..

[cit10] Gyun Shin I., Yeon Kim S., Moo Lee Y., Soo Cho C., Sung Y. K. (1998). J. Controlled Release.

[cit11] Zhou S., Deng X., Yang H. (2003). Biomaterials.

[cit12] He C., Sun J., Deng C., Zhao T., Deng M., Chen X., Jing X. (2004). Biomacromolecules.

[cit13] Gindy M. E., Ji S., Hoye T. R., Panagiotopoulos A. Z., Prud'Homme R. K. (2008). Biomacromolecules.

[cit14] Dash T. K., Konkimalla V. B. (2012). J. Controlled Release.

[cit15] Meerod S., Tumcharern G., Wichai U., Rutnakornpituk M. (2008). Polymer.

[cit16] Kim S. Y., Shin I. G., Lee Y. M., Cho C. S., Sung Y. K. (1998). J. Controlled Release.

[cit17] Bogdanov B., Vidts A., Van Den Bulcke A., Verbeeck R., Schacht E. (1998). Polymer.

[cit18] Pfefferkorn D., Pulst M., Naolou T., Busse K., Balko J., Kressler J. (2013). J. Polym. Sci., Part B: Polym. Phys..

[cit19] Wei X. W., Gong C. Y., Gou M. L., Fu S. Z., Guo Q. F., Shi S., Luo F., Guo G., Qiu L. Y., Qian Z. Y. (2009). Int. J. Pharm..

[cit20] Shen C., Guo S., Lu C. (2008). Polym. Adv. Technol..

[cit21] Lu C., Guo S. R., Zhang Y., Yin M. (2006). Polym. Int..

[cit22] Leroux F., Montembault V., Pascual S., Guerin W., Guillaume S. M., Fontaine L. (2014). Polym. Chem..

[cit23] Forrest M. L., Yáñez J. A., Remsberg C. M., Ohgami Y., Kwon G. S., Davies N. M. (2008). Pharm. Res..

[cit24] Mikhail A. S., Allen C. (2010). Biomacromolecules.

[cit25] Kim S. Y., Lee Y. M., Shin H. J., Kang J. S. (2001). Biomaterials.

[cit26] Suksiriworapong J., Sripha K., Kreuter J., Junyaprasert V. B. (2012). Int. J. Pharm..

[cit27] Drugbank, Indomethacin, http://www.drugbank.ca/drugs/DB00328, accessed 1 January 2016

[cit28] Cooper E. R. (2010). J. Controlled Release.

[cit29] Dong X., Zhang Z. M., Liu F., Wang W., Yu F., Lin Y. H., Jiang H. Y., He Z. Y. (2012). Acta Petrol. Sin..

[cit30] Saez A., Guzmán M., Molpeceres J., Aberturas M. R. (2000). Eur. J. Pharm. Biopharm..

[cit31] Merisko-Liversidge E. M., Liversidge G. G. (2008). Toxicol. Pathol..

[cit32] Zhang J., Wu L., Chan H. K., Watanabe W. (2011). Adv. Drug Delivery Rev..

[cit33] Saad W. S., Prud'Homme R. K. (2016). Nano Today.

[cit34] Zhang C., Long L., Xiong Y., Wang C., Peng C., Yuan Y., Liu Z., Lin Y., Jia Y., Zhou X., Li X. (2019). ACS Appl. Mater. Interfaces.

[cit35] Johnson B. K. , Flash NanoPrecipitation of Organic Actives via Confined Micromixing and Block Copolymer Stabilization, Princeton University, 2013

[cit36] Zhu Z. (2013). Biomaterials.

[cit37] Papadimitriou S., Bikiaris D. (2009). J. Controlled Release.

[cit38] Taresco V., Suksiriworapong J., Styliari I. D., Argent R. H., Swainson S. M. E. M. E. E., Booth J., Turpin E., Laughton C. A., Burley J. C., Alexander C., Garnett M. C. (2016). RSC Adv..

[cit39] D'Addio S. M., Prud'homme R. K. (2011). Adv. Drug Delivery Rev..

[cit40] Zhu Z. , Polymer Stabilized Nanosuspensions Formed via Flash Nanoprecipitation: Nanoparticle Formation, Formulation, and Stability, University of Minnesota, 2012

[cit41] Pustulka K. M., Wohl A. R., Lee H. S., Michel A. R., Han J., Hoye T. R., McCormick A. V., Panyam J., Macosko C. W. (2013). Mol. Pharm..

[cit42] Di Pasquale N., Marchisio D. L., Barresi A. A. (2012). Chem. Eng. Sci..

[cit43] Di Pasquale N., Marchisio D. L., Barresi A. A., Carbone P. (2014). J. Phys. Chem. B.

[cit44] Di Pasquale N., Marchisio D. L., Carbone P., Barresi A. A. (2013). Chem. Eng. Res. Des..

[cit45] Lince F., Marchisio D. L., Barresi A. A. (2008). J. Colloid Interface Sci..

[cit46] Mackenzie R., Booth J., Alexander C., Garnett M. C., Laughton C. A. (2015). J. Chem. Theory Comput..

[cit47] Huynh L., Grant J., Leroux J. C., Delmas P., Allen C. (2008). Pharm. Res..

[cit48] Costache A. D., Sheihet L., Zaveri K., Knight D. D., Kohn J. (2009). Mol. Pharm..

[cit49] Patel S. K., Lavasanifar A., Choi P. (2010). Biomaterials.

[cit50] Subashini M., Devarajan P. V., Sonavane G. S., Doble M. (2011). J. Mol. Model..

[cit51] Huynh L., Neale C., Pomès R., Allen C. (2010). Soft Matter.

[cit52] Taresco V., Gontrani L., Crisante F., Francolini I., Martinelli A., D'Ilario L., Bordi F., Piozzi A. (2015). J. Phys. Chem. B.

[cit53] Salerno K. M., Ismail A. E., Lane J. M. D., Grest G. S. (2014). J. Chem. Phys..

[cit54] Goliaei A., Lau E. Y., Adhikari U., Schwegler E., Berkowitz M. L. (2016). J. Phys. Chem. B.

[cit55] Spaeth J. R., Kevrekidis I. G., Panagiotopoulos A. Z. (2011). J. Chem. Phys..

[cit56] Giardiello M., Hatton F. L., Slater R. A., Chambon P., North J., Peacock A. K., He T., McDonald T. O., Owen A., Rannard S. P. (2016). Nanoscale.

[cit57] Abushrida A., Elhuni I., Taresco V., Marciani L., Stolnik S., Garnett M. C. (2020). J. Drug Delivery Sci. Technol..

[cit58] Malde A. K., Zuo L., Breeze M., Stroet M., Poger D., Nair P. C., Oostenbrink C., Mark A. E. (2011). J. Chem. Theory Comput..

[cit59] Koziara K. B., Stroet M., Malde A. K., Mark A. E. (2014). J. Comput.-Aided Mol. Des..

[cit60] Schmid N., Eichenberger A. P., Choutko A., Riniker S., Winger M., Mark A. E., Van Gunsteren W. F. (2011). Eur. Biophys. J..

[cit61] Geerke D. P., Van Gunsteren W. F. (2006). ChemPhysChem.

[cit62] Case D. A. , Belfon K., Ben-Shalom I. Y., Brozell S. R., Cerutti D. S., Cheatham III T. E., Cruzeiro V. W. D., Darden T. A., Duke R. E., Giambasu G., Gilson M. K., Gohlke H., Goetz A. W., Harris R., Izadi S., Kasavajhala K., Kovalenko A., Krasny R., Kurtzman T., Lee T. S., LeGrand S., Li P., Lin C., Liu J., Luchko T., Luo R., Man V., Merz K. M., Miao Y., Mikhailovskii O., Monard G., Nguyen H., Onufriev A., Pan F., Pantano S., Qi R., Roe D. R., Roitberg A., Sagui C., Schott-Verdugo S., Shen J., Simmerling C. L., Skrynnikov N., Smith J., Swails J., Walker R. C., Wang J., Wilson L., Wolf R. M., Wu X., York D. M. and Kollman P. A., AMBER 2018, University of California, 2018

[cit63] Martinez L., Andrade R., Birgin E. G., Martínez J. M. (2009). J. Comput. Chem..

[cit64] Berendsen H. J. C. , Postma J. P. M., van Gunsteren W. F. and Hermans J., in Intermolecular Forces: Proceedings of the Fourteenth Jerusalem Symposium on Quantum Chemistry and Biochemistry Held in Jerusalem, Israel, April 13–16, 1981, ed. B. Pullman, Springer Netherlands, Dordrecht, 1981, pp. 331–342

[cit65] Pronk S., Páll S., Schulz R., Larsson P., Bjelkmar P., Apostolov R., Shirts M. R., Smith J. C., Kasson P. M., Van Der Spoel D., Hess B., Lindahl E. (2013). Bioinformatics.

[cit66] U. N. S. Service , ARCHER, http://www.archer.ac.uk/

[cit67] Humphrey W., Dalke A., Schulten K. (1996). J. Mol. Graphics.

[cit68] Darden T., York D., Pedersen L. (1993). J. Chem. Phys..

[cit69] Essmann U., Perera L., Berkowitz M. L., Darden T., Lee H., Pedersen L. G. (1995). J. Chem. Phys..

[cit70] Kim M. S., Seo K. S., Khang G., Lee H. B. (2005). Macromol. Rapid Commun..

[cit71] Olivier A., Raquez J. M., Dubois P., Damman P. (2011). Eur. Polym. J..

[cit72] Simón L., Goodman J. M., Simon L., Goodman J. M., Simón L., Goodman J. M., Simo L., Goodman J. M., Simón L., Goodman J. M. (2007). J. Org. Chem..

[cit73] Ferrario M., Haughney M., McDonald I. R., Klein M. L. (1990). J. Chem. Phys..

[cit74] Jorgensen W. L., Briggs J. M., Leonor Contreras M. (1990). J. Phys. Chem..

[cit75] Kamath G., Georgiev G., Potoff J. J. (2005). J. Phys. Chem. B.

[cit76] Pereyra R. G., Asar M. L., Carignano M. A. (2011). Chem. Phys. Lett..

[cit77] Weerasinghe S., Smith P. E. (2003). J. Phys. Chem. B.

[cit78] Lavino A. D., Banetta L., Carbone P., Marchisio D. L. (2018). J. Phys. Chem. B.

[cit79] Jedlovszky P., Idrissi A., Jancsó G. (2009). J. Chem. Phys..

[cit80] Pinke A., Jedlovszky P. (2012). J. Phys. Chem. B.

[cit81] Taresco V., Suksiriworapong J., Creasey R., Burley J. C., Mantovani G., Alexander C., Treacher K., Booth J., Garnett M. C. (2016). J. Polym. Sci., Part A: Polym. Chem..

[cit82] Kumar V., Wang L., Riebe M., Tung H. H., Prud'homme R. K. (2009). Mol. Pharm..

[cit83] Kakde D., Taresco V., Bansal K. K., Magennis E. P., Howdle S. M., Mantovani G., Irvine D. J., Alexander C. (2016). J. Mater. Chem. B.

[cit84] Malvern , Malvern's introduction to zeta potential, http://www.malvern.com/en/support/resource-center/technical-notes/TN101104ZetaPotentialIntroduction.aspx

[cit85] Hondow N., Brydson R., Wang P., Holton M. D., Brown M. R., Rees P., Summers H. D., Brown A. (2012). J. Nanopart. Res..

[cit86] Kumar V., Prud'homme R. K. (2008). J. Pharm. Sci..

[cit87] Hüsler A., Haas S., Parry L., Romero M., Nisisako T., Williams P., Wildman R. D., Alexander M. R. (2018). RSC Adv..

[cit88] Matteucci M. E., Hotze M. A., Johnston K. P., Williams R. O. (2006). Langmuir.

[cit89] Han J., Zhu Z., Qian H., Wohl A. R., Beaman C. J., Hoye T. R., Macosko C. W. (2012). J. Pharm. Sci..

[cit90] D'Addio S. M., Kafka C., Akbulut M., Beattie P., Saad W., Herrera M., Kennedy M. T., Prud'Homme R. K. (2010). Mol. Pharm..

[cit91] de Oliveira A. M., Jäger E., Jäger A., Stepánek P., Giacomelli F. C. (2013). Colloids Surf., A.

[cit92] Yang L., Zhang Y., Cheng J., Yang C. (2019). J. Mol. Liq..

[cit93] Ramezanpour M., Leung S. S. W., Delgado-Magnero K. H., Bashe B. Y. M., Thewalt J., Tieleman D. P. (2016). Biochim. Biophys. Acta, Biomembr..

[cit94] Bally F., Garg D. K., Serra C. A., Hoarau Y., Anton N., Brochon C., Parida D., Vandamme T., Hadziioannou G. (2012). Polymer.

[cit95] Lince F., Marchisio D. L., Barresi A. A. (2011). Chem. Eng. Process..

