# Supplementary material for: Nanoformulation-by-design: an experimental and molecular dynamics study for polymer coated drug nanoparticles
Source: RSC Adv. 2020 May 21;10(33):19521–33. doi: 10.1039/d0ra00408a (PMC9054057; doi:10.1039/d0ra00408a)
Supplement: RA-010-D0RA00408A-s001 [file RA-010-D0RA00408A-s001.pdf]

Electronic Supplementary Material (ESI) for RSC Advances.
This journal is © The Royal Society of Chemistry 2020

Supporting Information for "Nanoformulation-by-design: An experimental and molecular dynamics study for polymer coated drug nanoparticles."

Contents

Table of Figures 2
Molecular Dynamics Section 5
Topologies and parameters for MD 5
Acetone 5
Polymer residual topologies 7
Control Simulation: mPEG350-PCL2000 in acetone 12
Control Simulation: mPEG350-PCL2000 in water 12
Control Simulation: Indomethacin NP in acetone 13
Control Simulation: Indomethacin NP in water 13
Control Simulation: Formation of polymeric NP in interfacial deposition 14
Control Simulation: Indomethacin NP in interfacial deposition 15
Main Simulation: Interfacial Deposition of mPEG350-PCL2000 16
Experimental Section 18
Polymer Characterisation: NMR 18
Polymer Characterisation: GPC 19
Polymer Characterisation: ATR-IR 23
Polymer Characterisation: DSC 23
Polymer Characterisation: Contact Angle 25
Polymer Characterisation: DSC 25
Polymeric NP Characterisation: DLS 27
Polymeric NP Characterisation: ζ-potential 27
Polymeric NP Characterisation TEM 28
Indomethacin bulk characterisation: DSC 29
Indomethacin NP Characterisation: DLS 29
Indomethacin NP Characterisation: POM 30
Polymer-coated indomethacin NP Characterisation: DLS 31
Polymer-coated indomethacin NP Characterisation: POM 33
Polymer-coated indomethacin NP Characterisation: TEM 34
Polymer-coated indomethacin NP Characterisation: Reproducibility 34
Polymer-coated indomethacin NP Characterisation: Stability 35
Polymer-coated indomethacin NP Characterisation: Effect of Drug amount 35
Polymer-coated indomethacin NP Characterisation: Drug Loading 36

Table of Figures

SI Figure 1: Five mPEG350-PCL2000 polymer chains surrounded by acetone (not shown), a favourable solvent. Periodic boundary conditions (PBC) apply, where the molecules can move across the end of the box. 12

SI Figure 2: Five mPEG350-PCL2000 polymer chains surrounded by water (not shown). The polymers self aggregate within 4ns and then they form a larger polymeric NP within the first 32 ns of the simulation. PBC apply.......... 12

SI Figure 3: A 5nm diameter indomethacin NP surrounded by acetone (not shown), a favourable solvent. The NP dissolves. PBC apply.......... 13

SI Figure 4: A 5nm diameter indomethacin NP surrounded by water (not shown). The NP stays intact. PBC apply. 13

SI Figure 5: mPEG350 (light blue)-b-PCL2000 (dark blue) polymer chains in the phase 1 of the interfacial deposition simulation, for the formation of a polymer NP. Snapshots taken at every 20 ns, solvents not shown for clarity. The polymer chains start to aggregate, 14

SI Figure 6: mPEG350 (light blue)-b-PCL2000 (dark blue) polymer chains in the phase 2 of the interfacial deposition simulation, for the formation of a polymer NP. Snapshots taken at the end of every 10 ns MD run where acetone molecules (pink) are replaced by water molecules. 14

SI Figure 7: Control simulation of the indomethacin NP (grey) to evaluate the NP's stability in the biphasic system. The drug NP stays intact until 70 ns, however as the presence acetone (pink) in the NP's surrounding environment increases, the NP starts to swell up. 15

SI Figure 8: Phase 1 of the interfacial deposition simulation of mPEG350 (light blue)-b-PCL2000 (dark blue) polymer chains in the presence of a 5 nm indomethacin NP (grey). Solvent molecules are not shown for clarity. PBC apply. Snapshots taken at various time points of the simulation. The polymer chains start to aggregate slowly as the acetone diffuses towards the water region. The indomethacin NP stays intact in the aqueous region until it reaches close proximity with the polymer chains and gets dissolved at 108 ns. . 16

SI Figure 9: Phase 2 of the interfacial deposition for the formation of a polymer-coated drug NP. Snapshots taken at the end of every 10 ns MD run where acetone molecules (pink) are replaced by water molecules, mimicking evaporation of the acetone. A polymer-drug NP of 7nm in diameter is formed. Water is not shown for clarity, PBC conditions apply. 16

SI Figure 10: Number of contacts and the distance between the PCL blocks and the indomethacin molecules during phase 1 of the interfacial deposition.......... 17

SI Figure 11: Radius of gyration (a, c) and end-to-end distances (b, d) for both phases 1 and 2 of the polymer-drug NP formation simulation. c and d refer just to phase 2....... 17

SI Figure 12: NMR Spectra of the synthesised polymers. PEG350PCL: 1H NMR (figure 4.1) (400 MHz, CDCl3, ppm): 1.41 (m,34, CH2CH2CH2), 1.68 (m, 68, CH2CH2CH2), 2.33 (t,

34, COCH2), 3.40 (s,3, PEGCH3), 3.67 (m, 24, OCH2CH2O), 4.08 (t, 34, CH2CH2CH2O), 4.25 (t,2, CH2CH2OCO). 18
SI Figure 13: GPC chromatograph of mPEG350PCL. 19
SI Figure 14: GPC chromatograph of mPEG550PCL. 20
SI Figure 15: GPC chromatograph of mPEG750PCL. 21
SI Figure 16: GPC chromatograph of mPEG2000PCL. 22
SI Figure 17: ATR IR spectra of the polymers. ATR-IR: u (cm-1) 3447, 2937, 2889, 2866, 1718, 1473, 1418, 1395, 1366, 1288, 1234, 1171, 1107, 1065, 1047, 957, 935, 733. 23
SI Figure 18: DSC of mPEG350PCL. 23
SI Figure 19: DSC of mPEG550PCL. 24
SI Figure 20: DSC of mPEG750PCL. 24
SI Figure 21: DSC of mPEG2000PCL. 24
SI Figure 22: Contact angle measurements of the 4 diblock copolymers for the assessment of their wettability properties. Error bars correspond to the standard deviation calculated from four measurements. The θ° values decrease with the increase of the hydrophilic block reflecting the increased hydrophilicity of the polymers. 25
SI Figure 23: Analysis of ΔH measurements from DSC revealed a decrease in the percentage of crystallinity of the PCL block of the copolymers with an increase of the length of the hydrophilic block. 25
SI Figure 24: Reproducibility of the interfacial deposition method; Overlay of size measurements (DLS) of 4 batches of polymer NPs formed with PEG350PCL at a 0.1 mg polymer amount. 27
SI Figure 25: Zeta potential of the polymeric NPs. Key refers to PEG chain length of polymer. 27
SI Figure 26: Stability of the PEG350PCL polymeric NPs (final polymer concentration in water 0.2 mg/ml): The original measurement is in red, the same sample after 1 month in green and the blue is after sonication of the latter for 5 minutes. The size distribution is the same in all 3 cases 28
SI Figure 27: TEM image of mPEG350- PCL 2000 polymeric NPs at 0.05 mg/ml initial concentration in acetone (polymer amount 1mg). Diameters of selected NP indicated. Scale bar 1000 nm. 28
SI Figure 28: DSC of indomethacin as received 29
SI Figure 29: Size distribution of Indomethacin in water (1mg/ml), just after sonication, by DLS. 29
SI Figure 30: Polarised optical microscopy images of indomethacin in aqueous phase prior to the coating experiments. (a) without and (b) with the polariser. Indomethacin is clearly crystalline. Magnification 10x. 30
SI Figure 31: mPEG350-PCL2000 coated indomethacin nanoparticle size distribution by DLS. (polymer amount 0.1mg) 31

SI Figure 32: Overlay of two experiments with mPEG350-PCL2000 and mPEG350-PCL2000 coated indomethacin nanoparticles (0.1mg polymer amount) to demonstrate the difference between the populations: Red line corresponds to the polymeric NPs that are formed in the absence of indomethacin, while the green distribution relates to the polymer-coated indomethacin 31
SI Figure 33: Centrifugation of the polymer coated drug nanoparticles (bottom graph) gives a PDI of 0.116, compared to a PDI that ranged from 0.394-0.265 in the pre-centrifugation suspensions (top graph)... 32
SI Figure 34: Polarised optical microscopy images of the mPEG350PCL-coated indomethacin NPs. (a1) and (a2) prior to centrifugation, where uncoated drug particles form aggregates and (b1), (b2) after the purification where no crystals were observed (Magnification 10x). 33
SI Figure 35: TEM picture of mPEG-PCL coated indomethacin particles, (highest polymer starting concentration). Scale bar 200nm. 34
SI Figure 36: Reproducibility of the coating method; Overlay of 5 batches of mPEG350PCL-coated indomethacin NPs (0.1 mg polymer amount, 1 mg/ml indomethacin concentration in water)... 34
SI Figure 37: Stability of the produced polymer coated-drug NPs; Same batch of mPEG350PCL-coated indomethacin NPs (0.1 mg polymer amount, 1 mg/ml indomethacin concentration in water) measured after 10 days. 35
SI Figure 38: Analysis of the effect of decreasing indomethacin concentration in the starting drug-bearing aqueous phase. Y-axis:Size distribution (Intensity), Right Y-axis PDI of measurements. 35

Molecular Dynamics Section

Topologies and parameters for MD

Acetone

```
;----------------------------TITLE -------------------------------------------
;   acetone
;
; This file was generated at 01:26 on 2014-09-24 by
;
;                  Automatic Topology Builder
;
;                   REVISION 2014-09-22
;-----------------------------------------------------------------------------
; Authors     : Alpeshkumar K. Malde, Le Zuo, Matthew Breeze, Martin Stroet, Alan
E. Mark
;
; Institute   : Molecular Dynamics group,
;               School of Chemistry and Molecular Biosciences (SCMB),
;               The University of Queensland, QLD 4072, Australia
; URL         : http://compbio.biosci.uq.edu.au/atb
; Citation    : Malde AK, Zuo L, Breeze M, Stroet M, Poger D, Nair PC, Oostenbrink
C, Mark AE.
;               An Automated force field Topology Builder (ATB) and repository:
version 1.0.
;               Journal of Chemical Theory and Computation, 2011, 7(12), 4026-
4037.
;               http://pubs.acs.org/doi/abs/10.1021/ct200196m
;
; Disclaimer  :
;      While every effort has been made to ensure the accuracy and validity of
parameters provided below
;      the assignment of parameters is being based on an automated procedure
combining data provided by a
;      given user as well as calculations performed using third party software.
They are provided as a guide.
;      The authors of the ATB cannot guarantee that the parameters are complete or
that the parameters provided
;      are appropriate for use in any specific application. Users are advised to
treat these parameters with discretion
;      and to perform additional validation tests for their specific application
if required. Neither the authors
;      of the ATB or The University of Queensland except any responsibly for how
the parameters may be used.
;
; Release notes and warnings:
;  (1) The topology is based on a set of atomic coordinates and other data
provided by the user after
;      after quantum mechanical optimization of the structure using different
levels of theory depending on
;      the nature of the molecule.
;  (2) In some cases the automatic bond, bond angle and dihedral type assignment
is ambiguous.
;      In these cases alternative type codes are provided at the end of the line.
;  (3) While bonded parameters are taken where possible from the nominated force
field non-standard bond, angle and dihedral
;      type code may be incorporated in cases where an exact match could not be
found. These are marked as "non-standard"
;      or "uncertain" in comments.
```

```
;  (4) In some cases it is not possible to assign an appropriate parameter
automatically. "%" is used as a place holder
;      for those fields that could not be determined automatically. The parameters
in these fields must be assigned manually
;      before the file can be used.
;------------------------------------------------------------------------------
; Input Structure : UAC
; Output          : UNITED ATOM topology
;       Use in conjunction with the corresponding united atom PDB file.
;-----------------------------------------------------------------------------
; Final Topology Generation was performed using:
; A B3LYP/6-31G* optimized geometry.
; Bonded and van der Waals parameters were taken from the GROMOS 53A6 parameter
set.
; Initial charges were estimated using the ESP method of Merz-Kollman.
; Final charges and charge groups were generated by method described in the ATB
paper.
; If required, additional bonded parameters were generated from a Hessian matrix
calculated at the B3LYP/6-31G* level of theory.
;
;       Topology file generated at 01:09 on 24 Sep 2014 for molecule
;       acetone (IUPAC: propan-2-one, database identifier: UAC)
;       by Automatic Topology Builder(revision 2014-09-22).
;
[ moleculetype ]
; Name   nrexcl
UAC     3
[ atoms ]
;  nr  type  resnr  resid  atom  cgnr  charge    mass    total_charge
    1   CH3    1    UAC     C3    1   -0.083  15.0350
    2     C    1    UAC     C2    1    0.734  12.0110
    3     O    1    UAC     O1    1   -0.568  15.9994      ;  0.083
    4   CH3    1    UAC     C1    2   -0.083  15.0350      ; -0.083
; total charge of the molecule:   0.000
[ bonds ]
;  ai   aj  funct   c0         c1
    1    2    2   0.1520   5.4300e+06
    2    3    2   0.1230   1.6600e+07
    2    4    2   0.1520   5.4300e+06
[ pairs ]
;  ai   aj  funct  ;  all 1-4 pairs but the ones excluded in GROMOS itp
[ angles ]
;  ai   aj   ak  funct   angle     fc
    1    2    3    2    125.00   750.00
    1    2    4    2    120.00   560.00
    3    2    4    2    125.00   750.00
[ dihedrals ]
; GROMOS improper dihedrals
;  ai   aj   ak   al  funct   angle     fc
    2    1    3    4    2      0.00   167.36
[ dihedrals ]
;  ai   aj   ak   al  funct    ph0      cp     mult
[ exclusions ]
;  ai   aj  funct  ;  GROMOS 1-4 exclusions
```

Polymer residual topologies

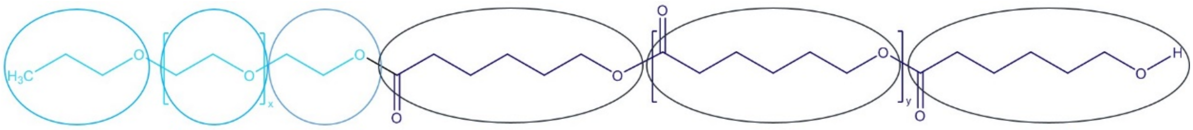


```
[ bondedtypes ]
; bonds  angles  dihedrals  impropers
    2       2          1          2

[ pgs ]
  [ atoms ]
;        name     type     charge   chargegroup
         C1       CH3      0.260    1
         OX       OE       -0.260   1


  [ bonds ]
;        ai       aj       gromos type
         C1       OX       gb_18    ;0.1430  8.1800e+06
         OX       +C1      gb_18    ;0.1430  8.1800e+06

  [ angles ]
;        ai       aj       ak       gromos type
         C1       OX       +C1      118.00      1080.00       ;109.50      320.00
         OX       +C1      +C2      ga_15

  [ dihedrals ]
;        ai       aj       ak       al       gromos type
         C1       OX       +C1      +C2      gd_23
         OX       +C1      +C2      +OX      gd_23


;The following is the REPEAT PEG part
;consists of -[CH2-CH2-O]-

[ pgr ]
[ atoms ]
;        name     type     charge   chargegroup
         C1       CH2      0.171    1
         C2       CH2      0.118    1
         OX       OE       -0.289   2

[ bonds ]
;        ai       aj       gromos type
         C1       C2       gb_27    ;0.1530  7.1500e+06
         C2       OX       gb_18
         OX       +C1      gb_18    ;for any of the next peg units

[ angles ]
;        ai       aj       ak       gromos type
         C1       C2       OX       ga_15            ;repeat unit
         C2       OX       +C1      118.00      1080.00               ;for the
repeat and the 1st CL unit
         OX       +C1      +C2      ga_15            ; for the repeat


 [ dihedrals ]
```

```
;        ai      aj      ak      al      gromos type
         C1      C2      OX      +C1     gd_23    ;for the next PEG and the 1st
PCL
         C2      OX      +C1     +C2     gd_23    ;for the next PEG
         OX      +C1     +C2     +OX     gd_23    ;for the PEG repeat CHANGED
IT FROM 37
         OX      +C1     +C2     +C3     gd_23    ;for the CL

[ pge ] ; we need this connecting site because the bonds and dihedrals in the
connection oxygen carbons are different from those on mpeg
[ atoms ]
;        name    type    charge   chargegroup
         C1      CH2     0.186    1
         C2      CH2     0.102    1
         OX      OE      -0.288   2

[ bonds ]
;        ai      aj      gromos type
         C1      C2      gb_27    ;0.1530  7.1500e+06
         C2      OX      gb_18
         OX      +C1     gb_5     ;for the CLs that is coming after

[ angles ]
;        ai      aj      ak      gromos type
         C1      C2      OX      ga_15    ; 111.00 530
         C2      OX      +C1     118.00     1080.00
         OX      +C1     +C2     ga_19
         OX      +C1     +OC     ga_33


 [ dihedrals ]
;        ai      aj      ak      al      gromos type
         C1      C2      OX      +C1     gd_12
         C2      OX      +C1     +C2     gd_13
         C2      OX      +C1     +OC     gd_12    ;for the CL carbonyl dihedral
         OX      +C1     +C2     +OC     gd_12    ;
         OX      +C1     +C2     +C3     gd_12
[ impropers ]

         OX      +C1     +OC     +C2     gi_1     ;0.00    167.36   ;for the
connection with the CL unit

;Caprolactone connecting unit
[ pcs ]
[ atoms ]
;        name    type    charge   chargegroup
         C1      C       0.226    1
         OC      O       -0.352   1
         C2      CH2     0.085    1
         C3      CH2     0.041    2
         C4      CH2     0.000    2
         C5      CH2     0.028    3
         C6      CH2     0.254    3
         OX      OA      -0.282   3

[ bonds ]
;        ai      aj      gromos type
         C1      OC      gb_5
         C1      C2      gb_27
         C2      C3      gb_27
         C3      C4      gb_27
         C4      C5      gb_27
```

```
        C5      C6      gb_27
        C6      OX      gb_18
        OX      +C1     gb_18   ;for the repeat residue

[ angles ]
;       ai      aj      ak      gromos type
        OC      C1      C2      ga_30   ;121.00     685.00
        C1      C2      C3      ga_15   ;111.00     530.00
        C2      C3      C4      ga_15
        C3      C4      C5      ga_15
        C4      C5      C6      ga_15
        C5      C6      OX      ga_13   ;109.50     520.00
        C6      OX      +C1     118.00      1080.00
        OX      +C1     +C2     ga_30
        OX      +C1     +OC     ga_33


[ dihedrals ]
;       ai      aj      ak      al      gromos type
        OC      C1      C2      C3      gd_12   ;
        C1      C2      C3      C4      gd_29   ;0.00     3.77    3
        C2      C3      C4      C5      gd_29   ;0.00     3.77    3
        C3      C4      C5      C6      gd_29   ;0.00     3.77    3
        C4      C5      C6      OX      gd_23   ;
        C5      C6      OX      +C1     gd_12   ;
        C6      OX      +C1     +C2     gd_13   ;for the repeat residue
        C6      OX      +C1     +OC     gd_12   ;repeat residue
        OX      +C1     +C2     +C3     gd_12   ;repeat residue

[ impropers ]

        OX      +C1     +OC     +C2     gi_1    ;0.00   167.36

; CAPROLACTONE  UNITED atom
; Constructed by Ioanna

;The following is the cl monomer residue
;consists of -[O=C-CH2-CH2-CH2-CH2-CH2-0]
[ pcl ]
[ atoms ]
;       name    type    charge  chargegroup
        C1      C       0.271   1
        OC      O       -0.352  1
        C2      CH2     0.081   1
        C3      CH2     -0.013  2
        C4      CH2     0.013   2
        C5      CH2     0.027   3
        C6      CH2     0.255   3
        OX      OA      -0.282  3

[ bonds ]
;       ai      aj      gromos type
        C1      OC      gb_5
        C1      C2      gb_27
        C2      C3      gb_27
        C3      C4      gb_27
        C4      C5      gb_27
        C5      C6      gb_27
        C6      OX      gb_18
        OX      +C1     gb_18   ;for the repeat residue

[ angles ]
```

```
;       ai      aj      ak      gromos type
        OC      C1      C2      ga_30   ;121.00     685.00
        C1      C2      C3      ga_15   ;111.00     530.00
        C2      C3      C4      ga_15
        C3      C4      C5      ga_15
        C4      C5      C6      ga_15
        C5      C6      OX      ga_13   ;109.50     520.00
        C6      OX      +C1     118.00     1080.00
        OX      +C1     +C2     ga_30
        OX      +C1     +OC     ga_33


[ dihedrals ]
;       ai      aj      ak      al      gromos type
        OC      C1      C2      C3      gd_12   ;
        C1      C2      C3      C4      gd_29   ;0.00     3.77    3
        C2      C3      C4      C5      gd_29   ;0.00     3.77    3
        C3      C4      C5      C6      gd_29   ;0.00     3.77    3
        C4      C5      C6      OX      gd_23   ;
        C5      C6      OX      +C1     gd_12   ;
        C6      OX      +C1     +C2     gd_13   ;for the repeat residue
        C6      OX      +C1     +OC     gd_12   ;repeat residue
        OX      +C1     +C2     +C3     gd_12   ;repeat residue

[ impropers ]

        OX      +C1     +OC     +C2     gi_1    ;0.00    167.36

[ pce]
[ atoms ]
;       name    type    charge  chargegroup
        C1      C       0.234   1
        OC      O       -0.353  1
        C2      CH2     0.078   1
        C3      CH2     0.041   1
        C4      CH2     0.000   2
        C5      CH2     0.033   2
        C6      CH2     0.100   3
        OX      OA      -0.328  3
        HA      H       0.195   3


[ bonds ]
;       ai      aj      gromos type
        C1      OC      gb_5
        C1      C2      gb_27
        C2      C3      gb_27
        C3      C4      gb_27
        C4      C5      gb_27
        C5      C6      gb_27
        C6      OX      gb_18
        OX      HA      gb_1

[ angles ]
;       ai      aj      ak      gromos type
        OC      C1      C2      ga_30   ;121.00     685.00
        C1      C2      C3      ga_15   ;111.00     530.00
        C2      C3      C4      ga_15
        C3      C4      C5      ga_15
        C4      C5      C6      ga_15
        C5      C6      OX      ga_13   ;109.50     520.00
        C6      OX      HA      118.00     1080.00
```

```
[ dihedrals ]
;       ai      aj      ak      al      gromos type
        OC      C1      C2      C3      gd_12   ;
        C1      C2      C3      C4      gd_29   ;0.00     3.77    3
        C2      C3      C4      C5      gd_29   ;0.00     3.77    3
        C3      C4      C5      C6      gd_29   ;0.00     3.77    3
        C4      C5      C6      OX      gd_23   ;
        C5      C6      OX      HA      gd_30   ;

;       H1      N1      C1      C2
        N1      C1      O1      C2      2      0.00   167.36
;       N1      C1      C2      H2
;       N1      C1      C2      +N1
;       C1      C2      H2      +N1
;       C1      C2      H2      C3
;       C1      C2      C3      C4
;       C1      C2      +N1     +C1
;       C2      H2      C3      C4
        C2      C3      C4      C5      1      0.00     5.92    3
;       C2      H2      +N1     +H1
;       C2      H2      +N1     +C1
;       C2      +N1     +H1     +C1
        C2      +N1     +C1     +O1     1    180.00    33.50    2
;       C2      +N1     +C1     +C2
        C3      C4      C5      02      1    180.00     1.00    6
;       C3      C4      C5      03
;       C4      C5      O3      H3
        O2      C5      O3      H3      1    180.00     7.11    2
```

Control Simulation: mPEG350-PCL2000 in acetone

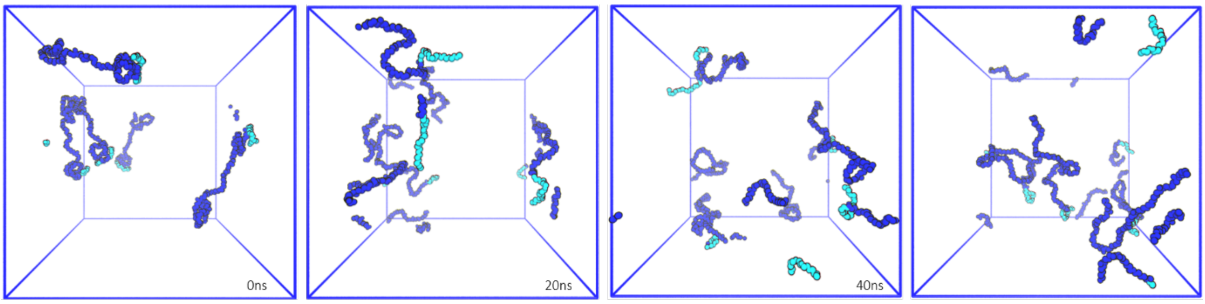

SI Figure 1: Five mPEG350-PCL2000 polymer chains surrounded by acetone (not shown), a favourable solvent. Periodic boundary conditions (PBC) apply, where the molecules can move across the end of the box.

Control Simulation: mPEG350-PCL2000 in water

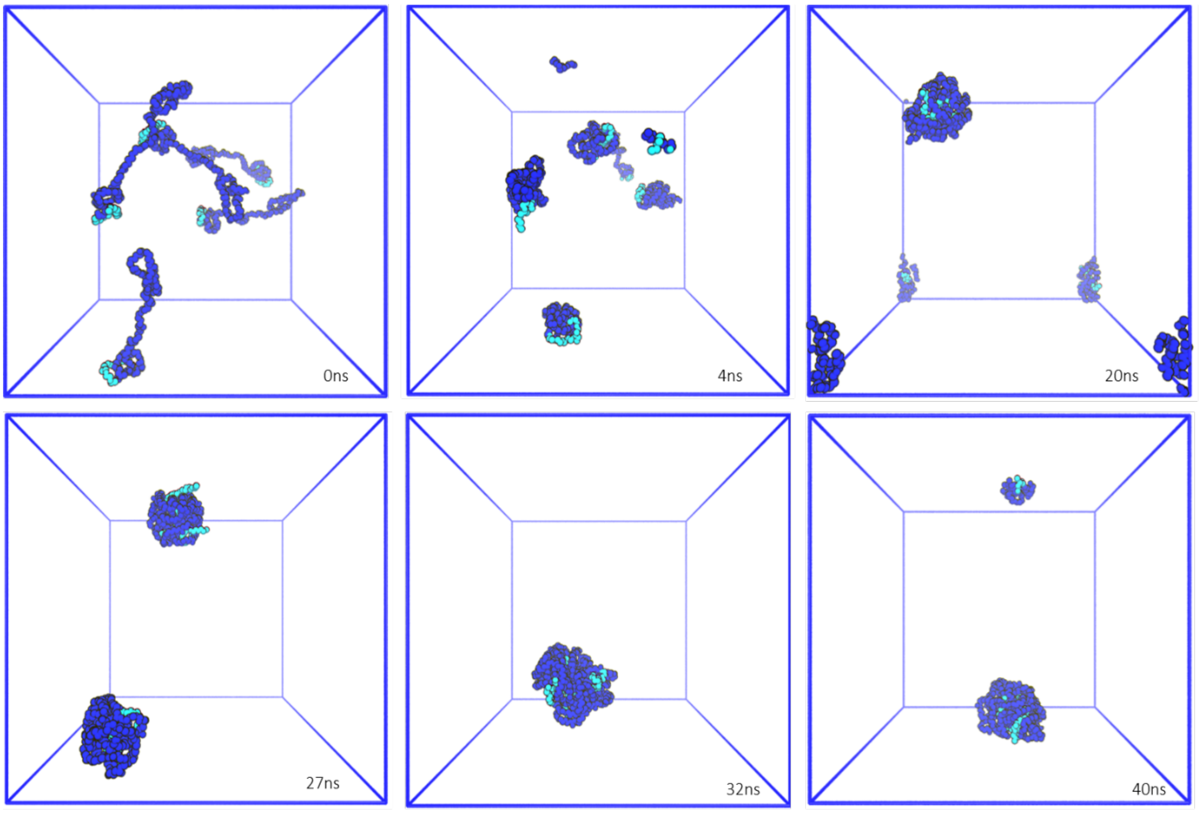

SI Figure 2: Five mPEG350-PCL2000 polymer chains surrounded by water (not shown). The polymers self aggregate within 4ns and then they form a larger polymeric NP within the first 32 ns of the simulation. PBC apply.

Control Simulation: Indomethacin NP in acetone

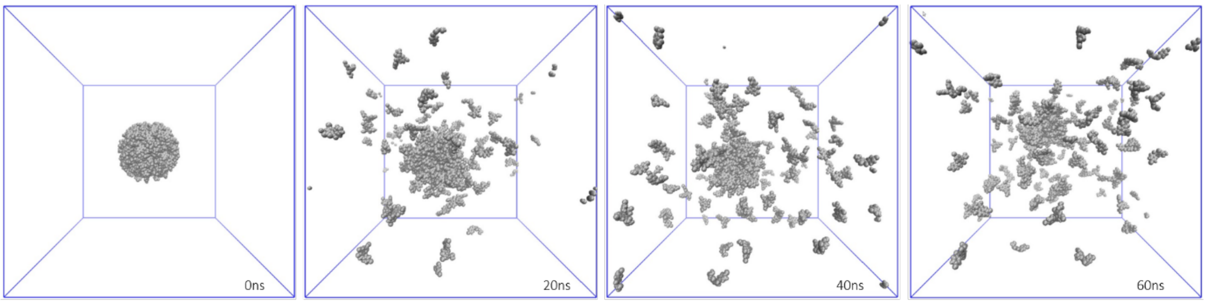

SI Figure 3: A 5nm diameter indomethacin NP surrounded by acetone (not shown), a favourable solvent. The NP dissolves. PBC apply.

Control Simulation: Indomethacin NP in water

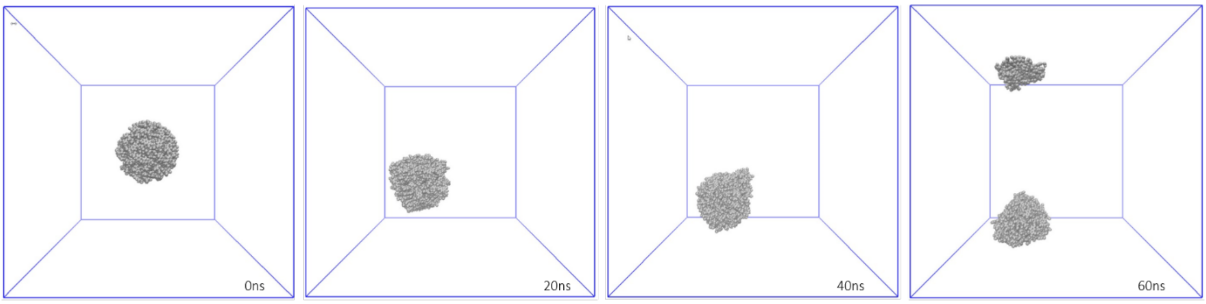

SI Figure 4: A 5nm diameter indomethacin NP surrounded by water (not shown). The NP stays intact. PBC apply.

Control Simulation: Formation of polymeric NP in interfacial deposition

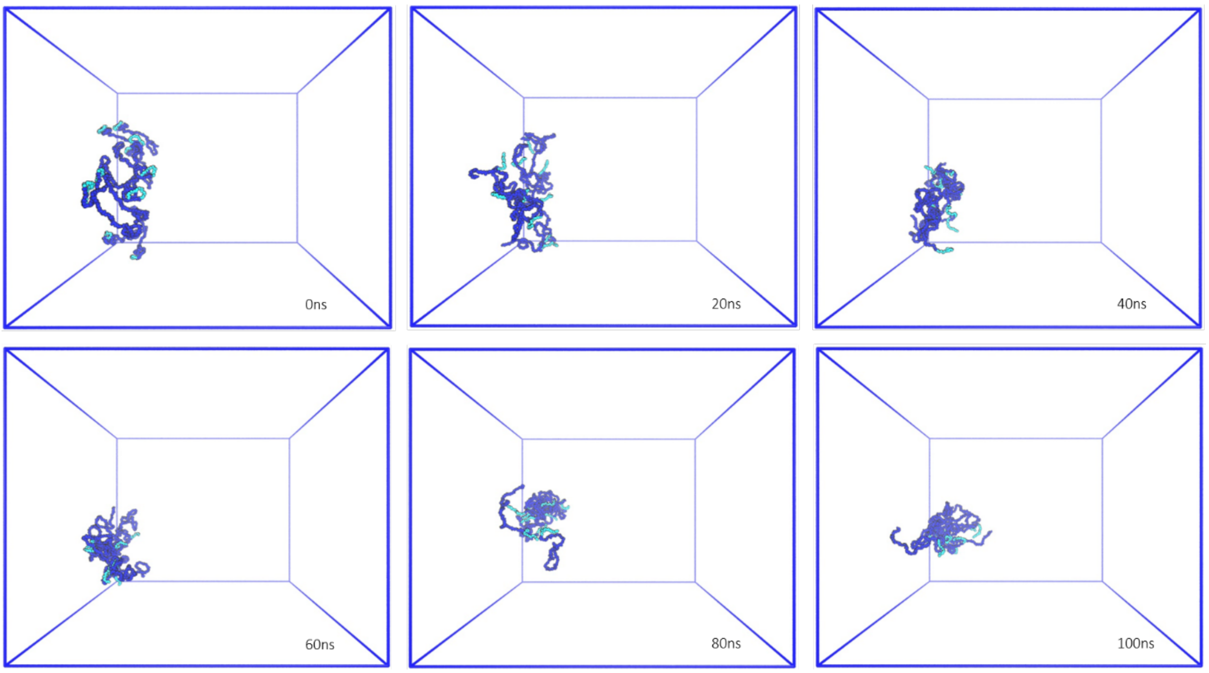

SI Figure 5: mPEG350 (light blue)-b-PCL2000 (dark blue) polymer chains in the phase 1 of the interfacial deposition simulation, for the formation of a polymer NP. Snapshots taken at every 20 ns, solvents not shown for clarity. The polymer chains start to aggregate,

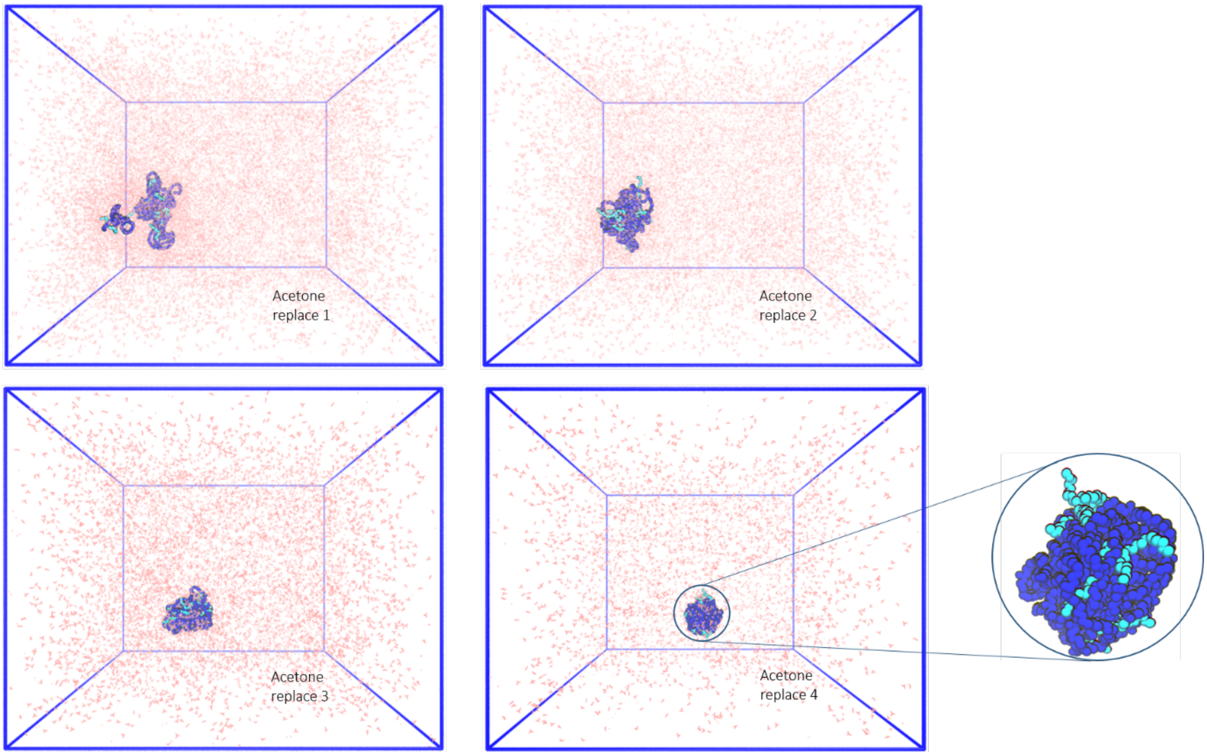

SI Figure 6: mPEG350 (light blue)-b-PCL2000 (dark blue) polymer chains in the phase 2 of the interfacial deposition simulation, for the formation of a polymer NP. Snapshots taken at the end of every 10 ns MD run where acetone molecules (pink) are replaced by water molecules.

Control Simulation: Indomethacin NP in interfacial deposition

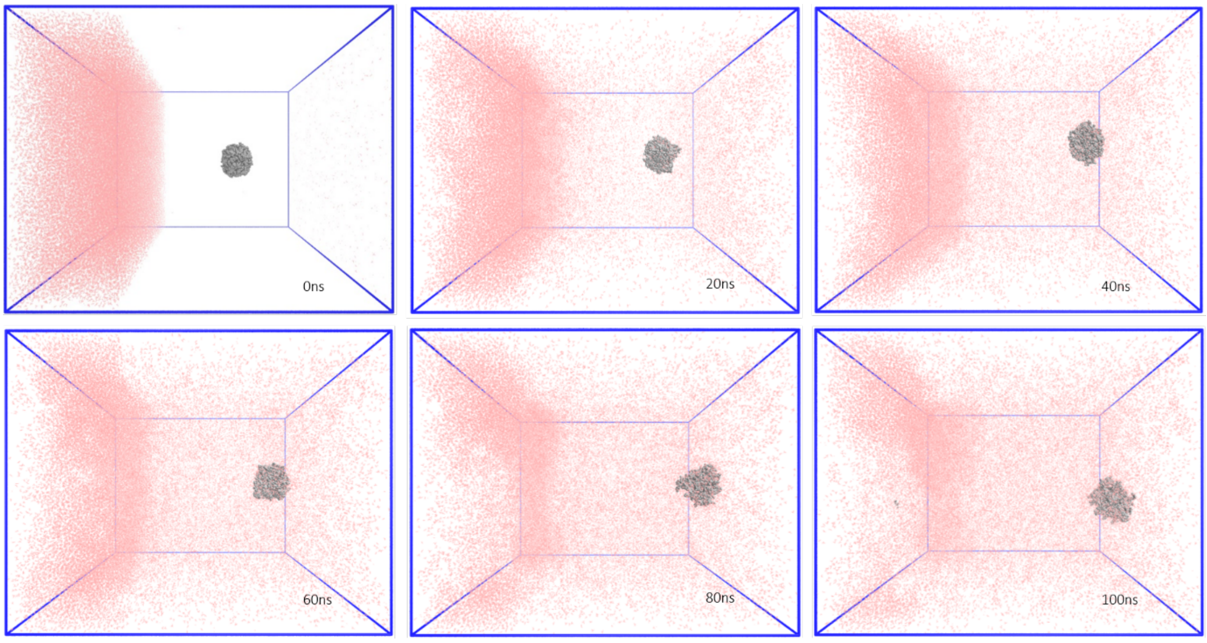

SI Figure 7: Control simulation of the indomethacin NP (grey) to evaluate the NP's stability in the biphasic system. The drug NP stays intact until 70 ns, however as the presence acetone (pink) in the NP's surrounding environment increases, the NP starts to swell up.

Main Simulation: Interfacial Deposition of mPEG350-PCL2000

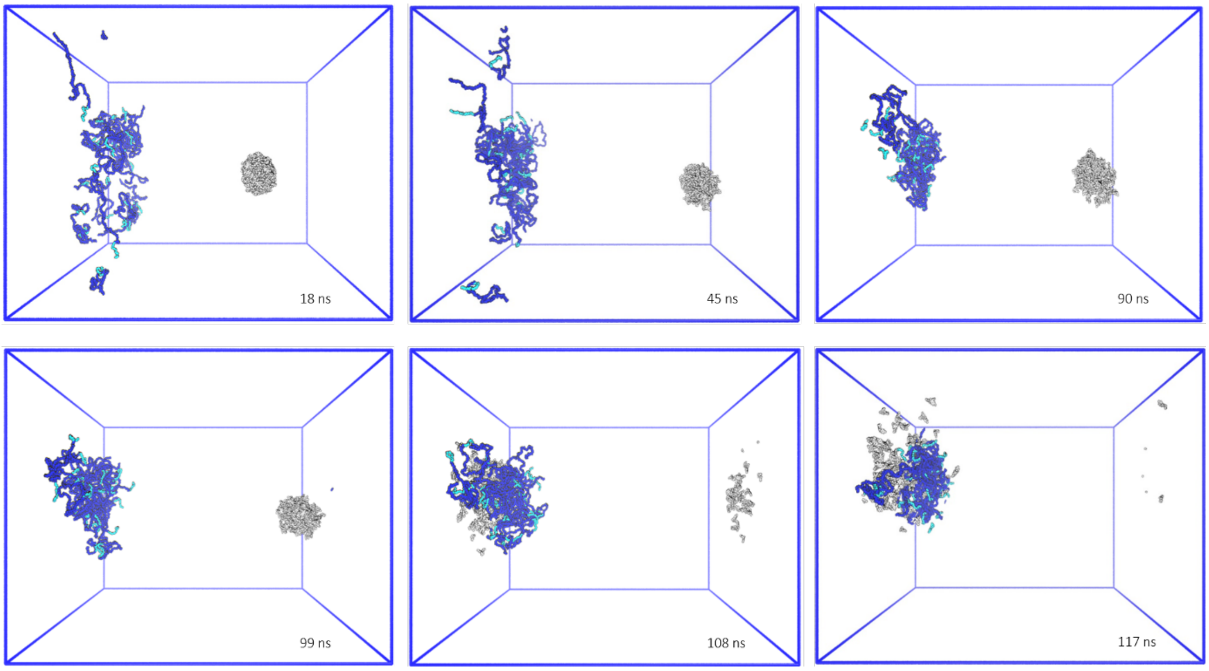

SI Figure 8: Phase 1 of the interfacial deposition simulation of mPEG350 (light blue)-b-PCL2000 (dark blue) polymer chains in the presence of a 5 nm indomethacin NP (grey). Solvent molecules are not shown for clarity. PBC apply. Snapshots taken at various time points of the simulation. The polymer chains start to aggregate slowly as the acetone diffuses towards the water region. The indomethacin NP stays intact in the aqueous region until it reaches close proximity with the polymer chains and gets dissolved at 108 ns.

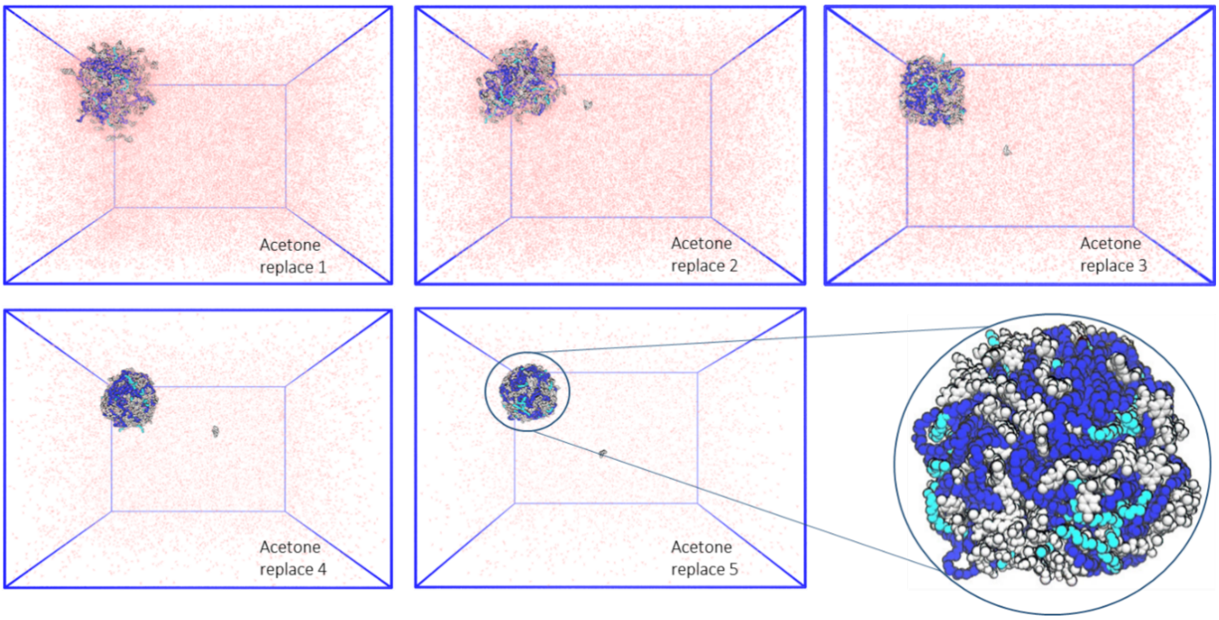

SI Figure 9: Phase 2 of the interfacial deposition for the formation of a polymer-coated drug NP. Snapshots taken at the end of every 10 ns MD run where acetone molecules (pink) are replaced by water molecules, mimicking evaporation of the acetone. A polymer-drug NP of 7nm in diameter is formed. Water is not shown for clarity, PBC conditions apply.

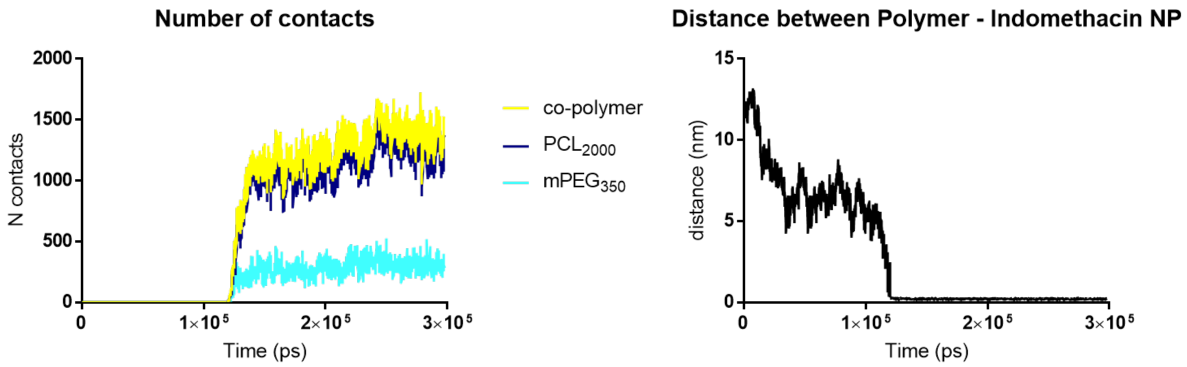

SI Figure 10: Number of contacts and the distance between the PCL blocks and the indomethacin molecules during phase 1 of the interfacial deposition.

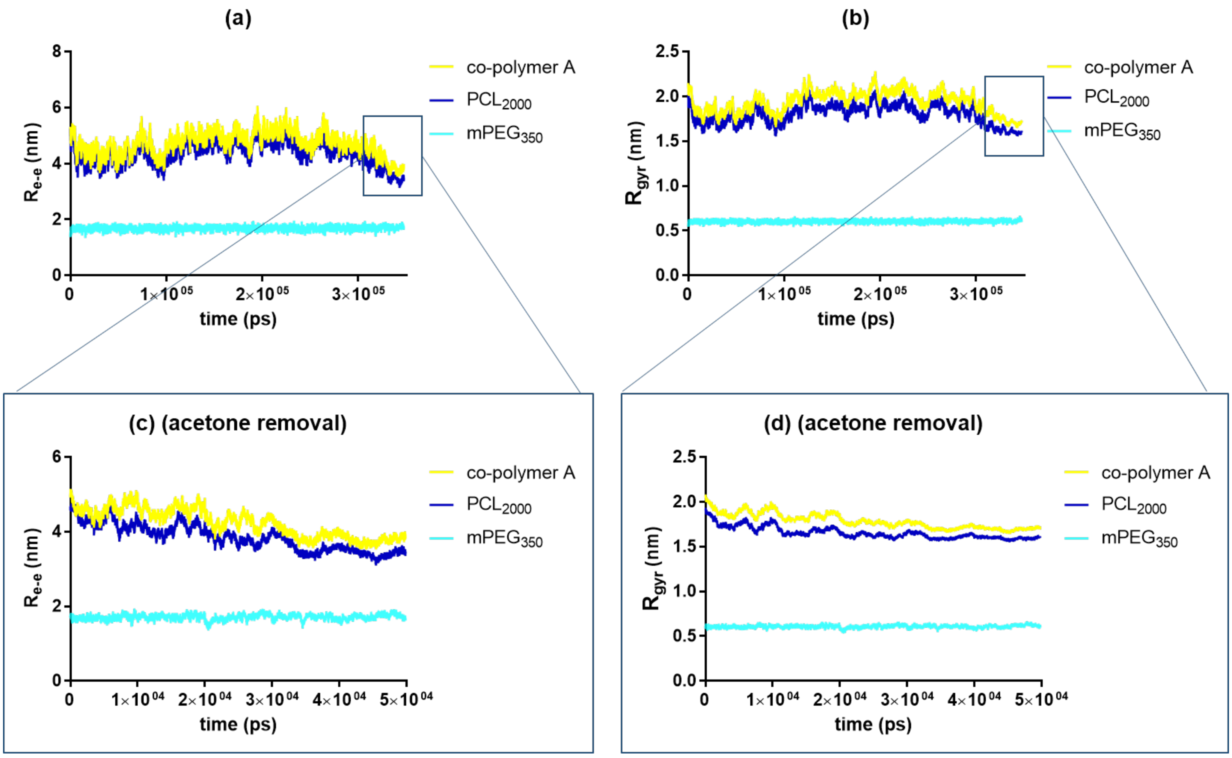

SI Figure 11: Radius of gyration (a, c) and end-to-end distances (b, d) for both phases 1 and 2 of the polymer-drug NP formation simulation. c and d refer just to phase 2.

Experimental Section

Polymer Characterisation: NMR

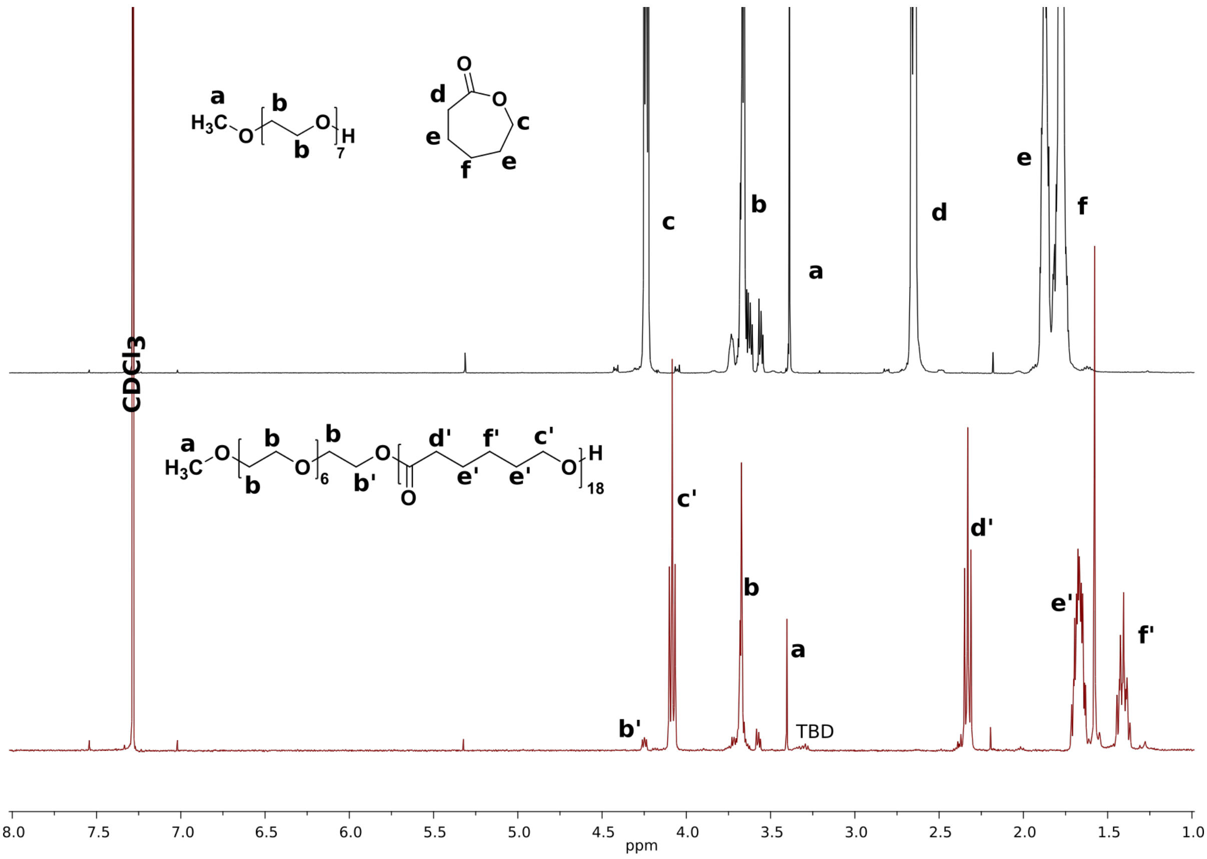

SI Figure 12: NMR Spectra of the synthesised polymers. PEG350PCL: 1H NMR (figure 4.1) (400 MHz, CDCl3, ppm): 1.41 (m,34, CH2CH2CH2), 1.68 (m, 68, CH2CH2CH2), 2.33 (t, 34, COCH2), 3.40 (s,3, PEGCH3), 3.67 (m, 24, OCH2CH2O), 4.08 (t, 34, CH2CH2CH2O), 4.25 (t,2, CH2CH2OCO).

Polymer Characterisation: GPC

Sample:IDSA09 **Date:24/04/2015 17:32:03**

Workbook: CHCl3_conv_150420.plw Inj File: 150424-0006.cgrm
Path: C:\Cirrus Workbooks\2015\CHCl3_conv_150420\CHCl3_conv_150420.plw
Res. File: 150424-0006.rst
Method: PSeasy_CHCl3_150420 Batch: 150424
Eluent: Chloroform

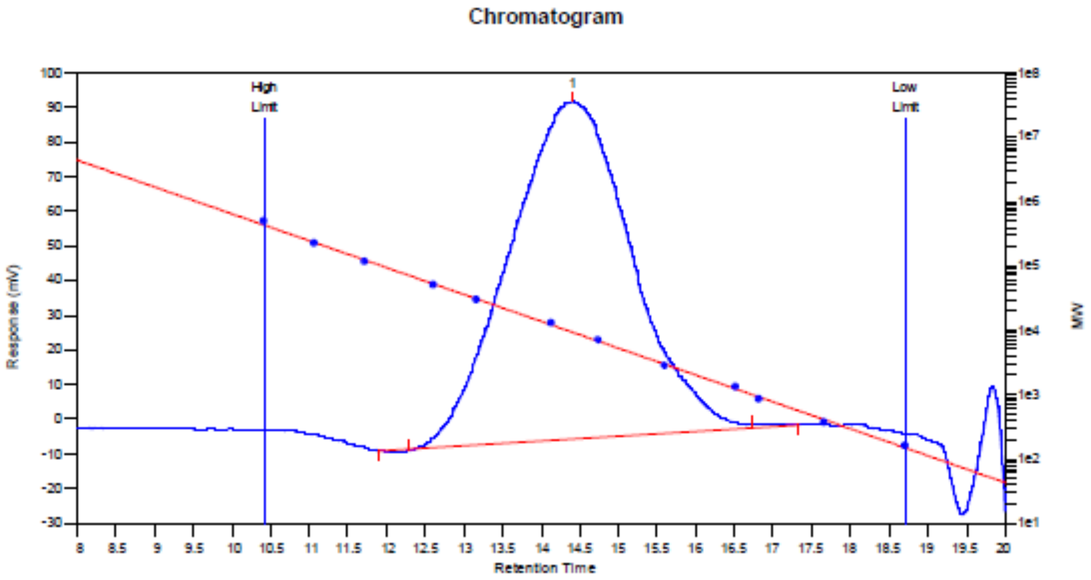

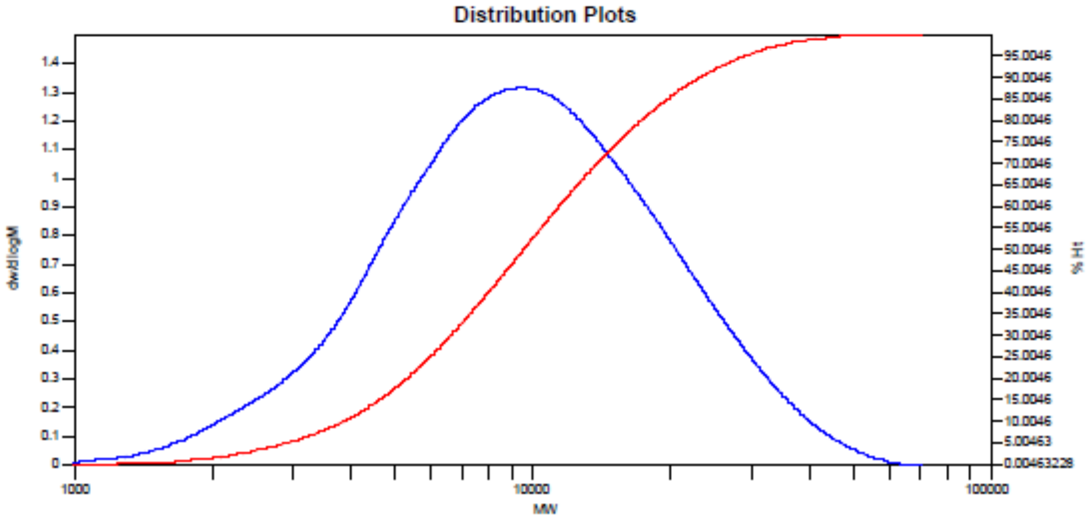

Peak No	Mp	Mn	Mw	Mz	Mz+1	Mv	PD
1	9447	7384	11839	17751	24269	11081	1.60333

SI Figure 13: GPC chromatograph of mPEG350PCL.

Sample:IDSB10 **Date:24/04/2015 15:44:17**

Workbook: CHCl3_conv_150420.plw Inj File: 150424-0003.cgrm
Path: C:\Cirrus Workbooks\2015\CHCl3_conv_150420\CHCl3_conv_150420.plw
Res. File: 150424-0003-Repeat (01).rst
Method: PSeasy_CHCl3_150420 Batch: 150424
Eluent: Chloroform

Chromatogram

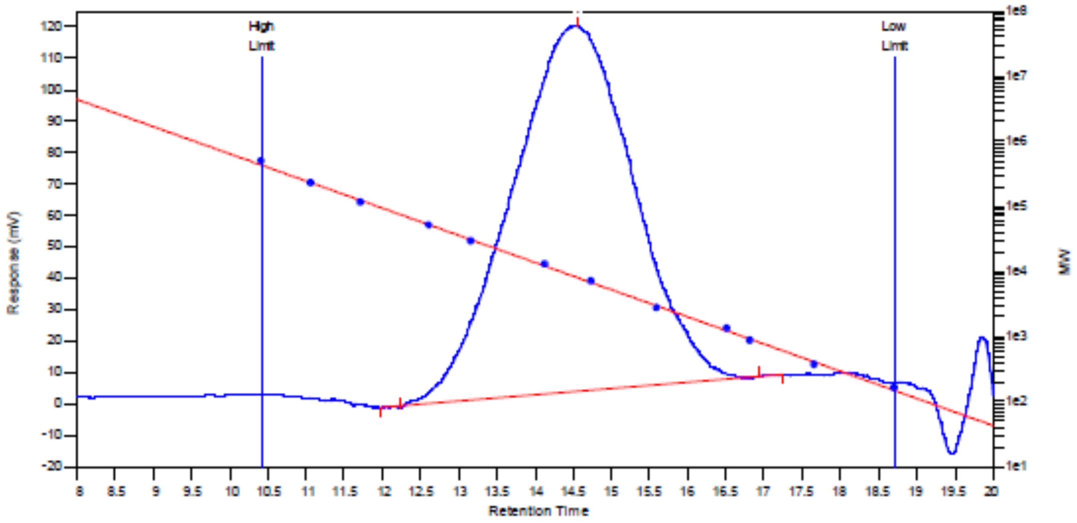

Distribution Plots

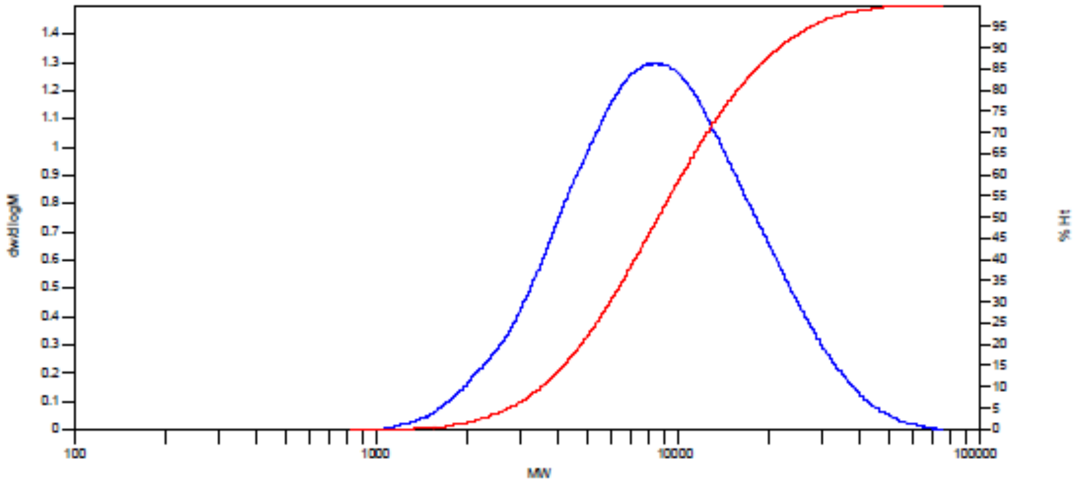

Peak No	Mp	Mn	Mw	Mz	Mz+1	Mv	PD
1	8790	6823	10966	17114	24611	10219	1.60721

SI Figure 14: GPC chromatograph of mPEG550PCL.

Sample:IDSC11 **Date:24/04/2015 16:56:13**

Workbook: CHCl3_conv_150420.plw Inj File: 150424-0005.cgrm
Path: C:\Cirrus Workbooks\2015\CHCl3_conv_150420\CHCl3_conv_150420.plw
Res. File: 150424-0005-Repeat (01).rst
Method: PSeasy_CHCl3_150420 Batch: 150424
Eluent: Chloroform

Chromatogram

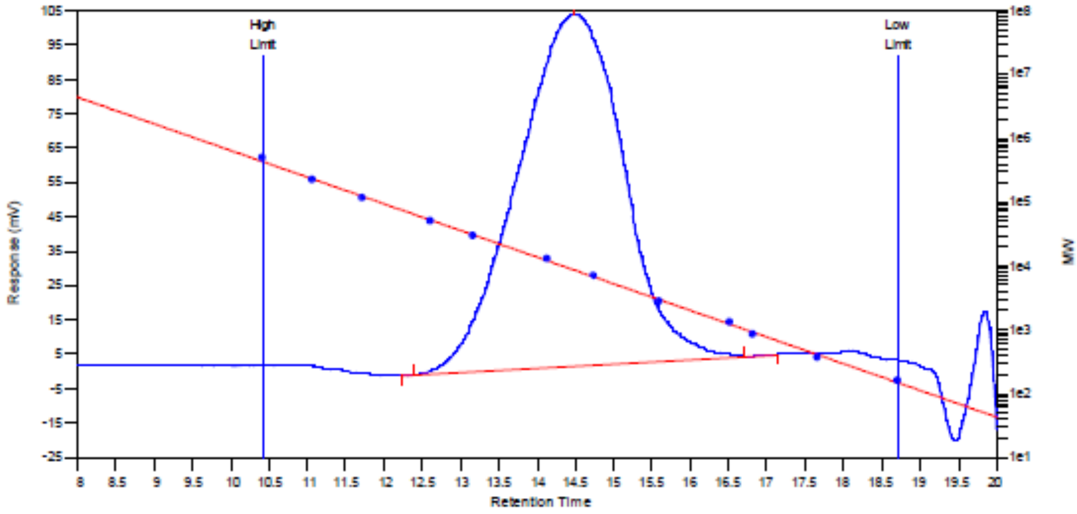

Distribution Plots

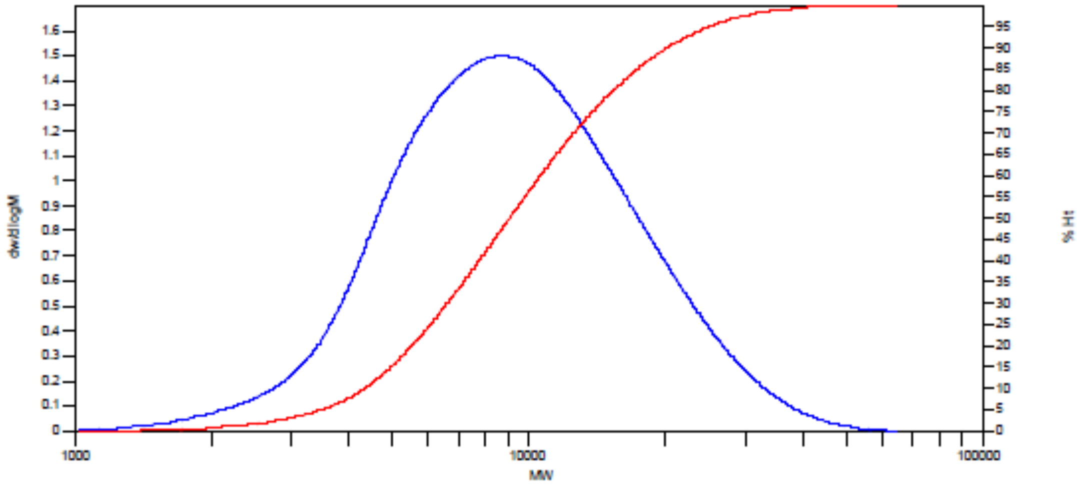

Peak No	Mp	Mn	Mw	Mz	Mz+1	Mv	PD
1	8678	7601	10911	15380	20763	10345	1.43547

SI Figure 15: GPC chromatograph of mPEG750PCL.

Sample:IDSF16 **Date:24/04/2015 15:08:27**

Workbook: CHCl3_conv_150420.plw Inj File: 150424-0002.cgrm
Path: C:\Cirrus Workbooks\2015\CHCl3_conv_150420\CHCl3_conv_150420.plw
Res. File: 150424-0002-Repeat (01).rst
Method: PSeasy_CHCl3_150420 Batch: 150424
Eluent: Chloroform

Chromatogram

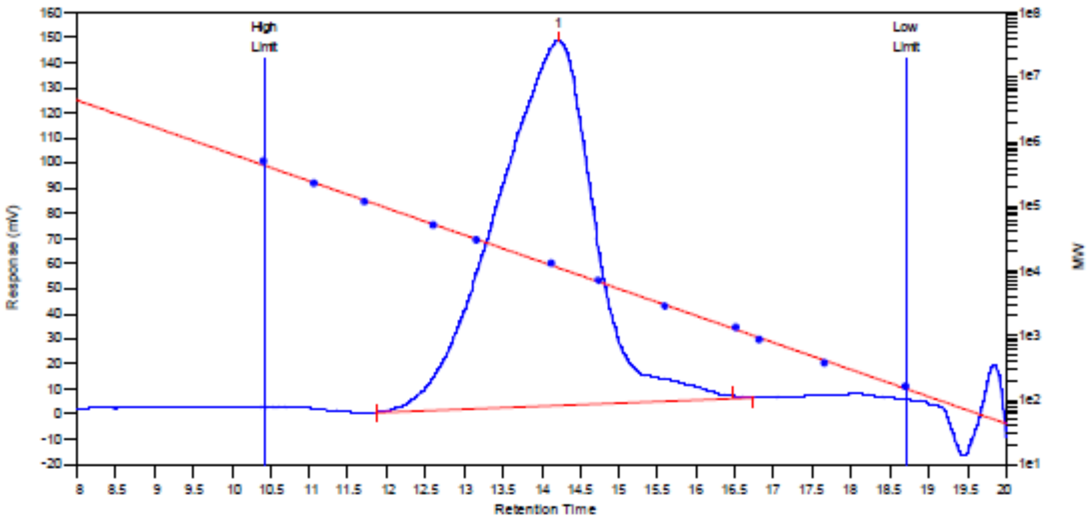

Distribution Plots

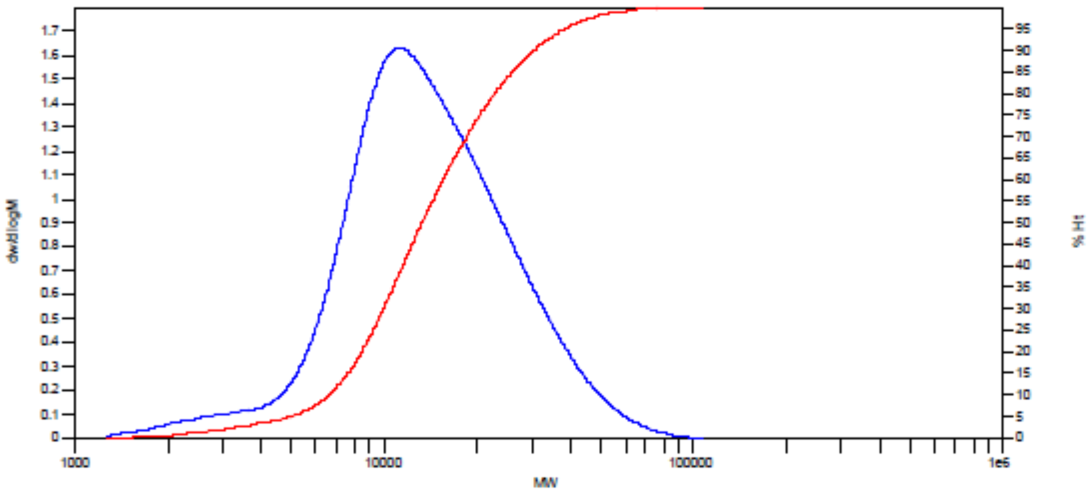

Peak No	Mp	Mn	Mw	Mz	Mz+1	Mv	PD
1	11268	10957	16353	23604	32805	15456	1.49247

SI Figure 16: GPC chromatograph of mPEG2000PCL.

Polymer Characterisation: ATR-IR

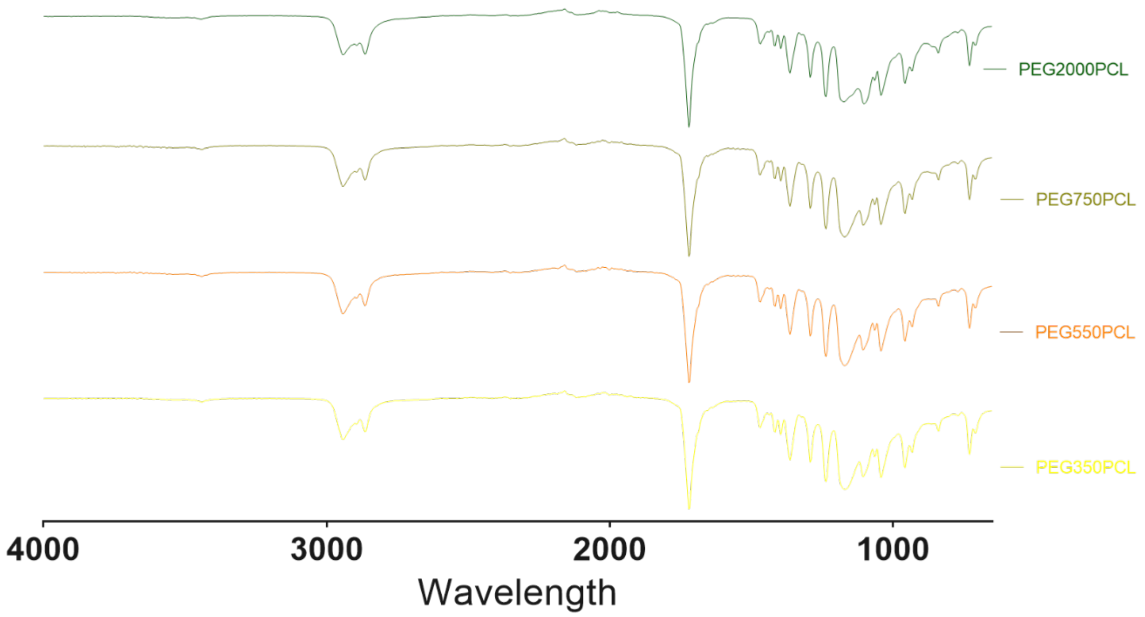

SI Figure 17: ATR IR spectra of the polymers. ATR-IR: u (cm-1) 3447, 2937, 2889, 2866, 1718, 1473, 1418, 1395, 1366, 1288, 1234, 1171, 1107, 1065, 1047, 957, 935, 733.

Polymer Characterisation: DSC

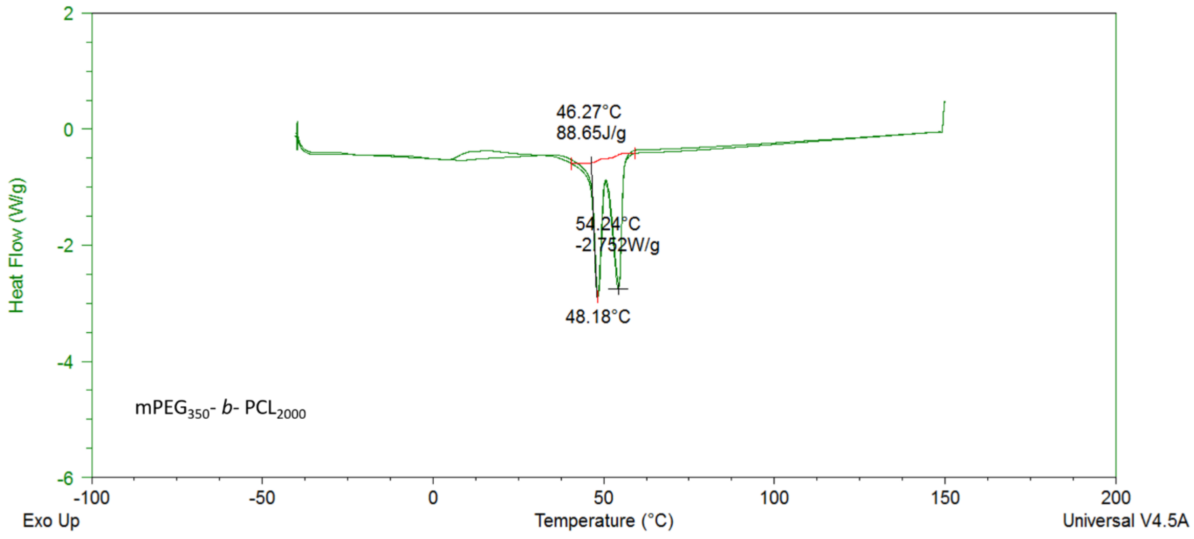

SI Figure 18: DSC of mPEG350PCL.

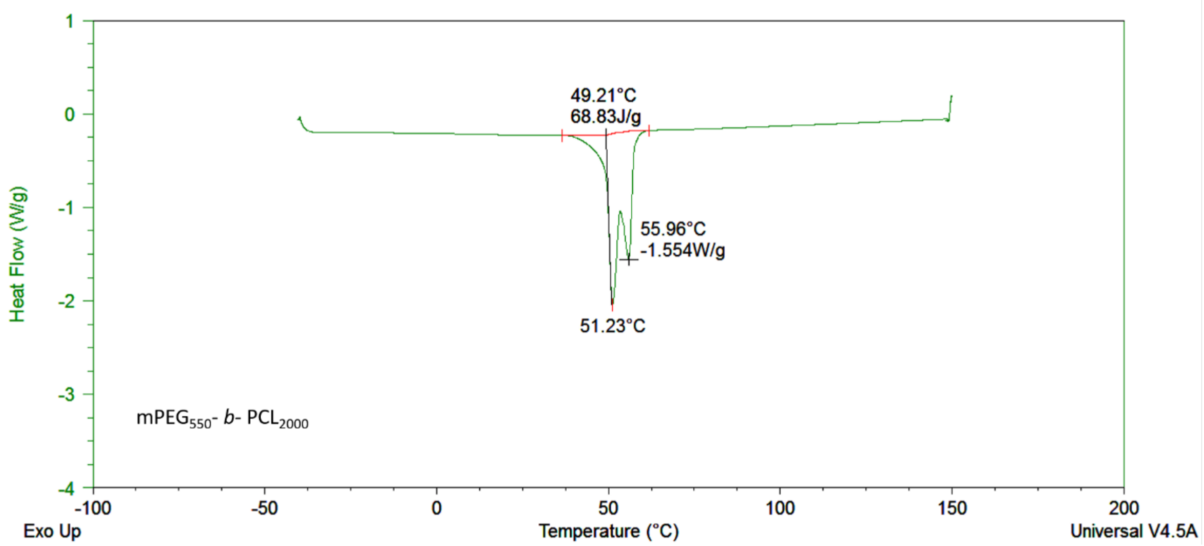

SI Figure 19: DSC of mPEG550PCL.

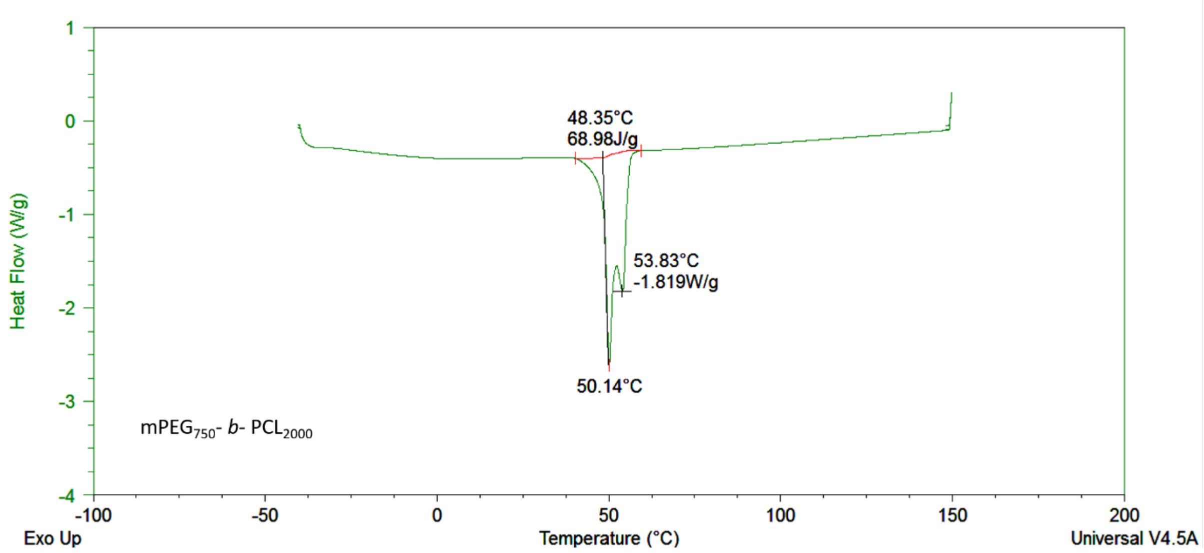

SI Figure 20: DSC of mPEG750PCL.

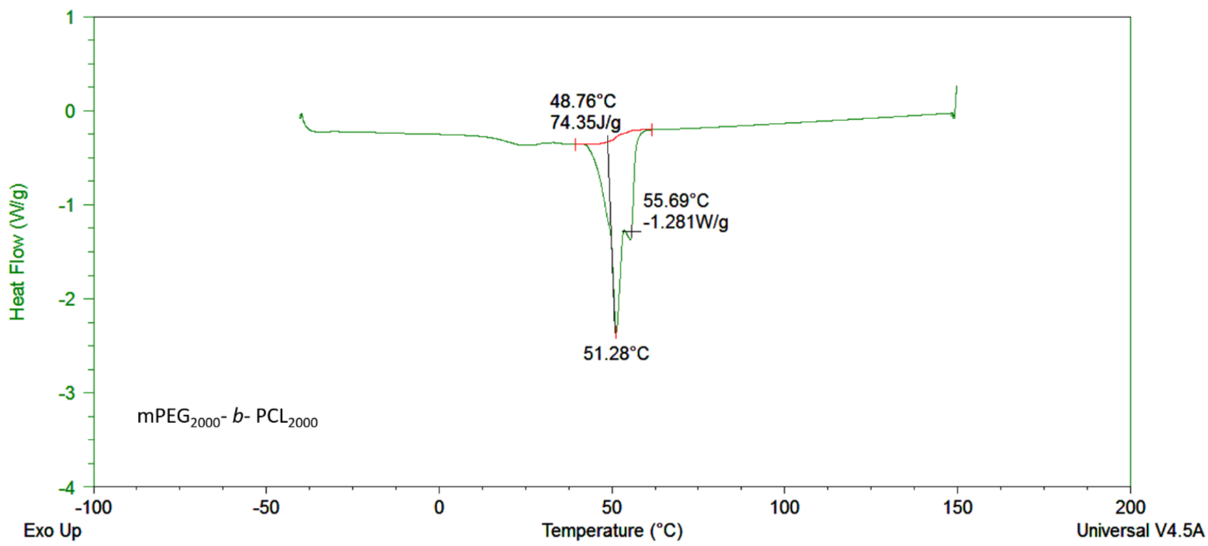

SI Figure 21: DSC of mPEG2000PCL.

Polymer Characterisation: Contact Angle

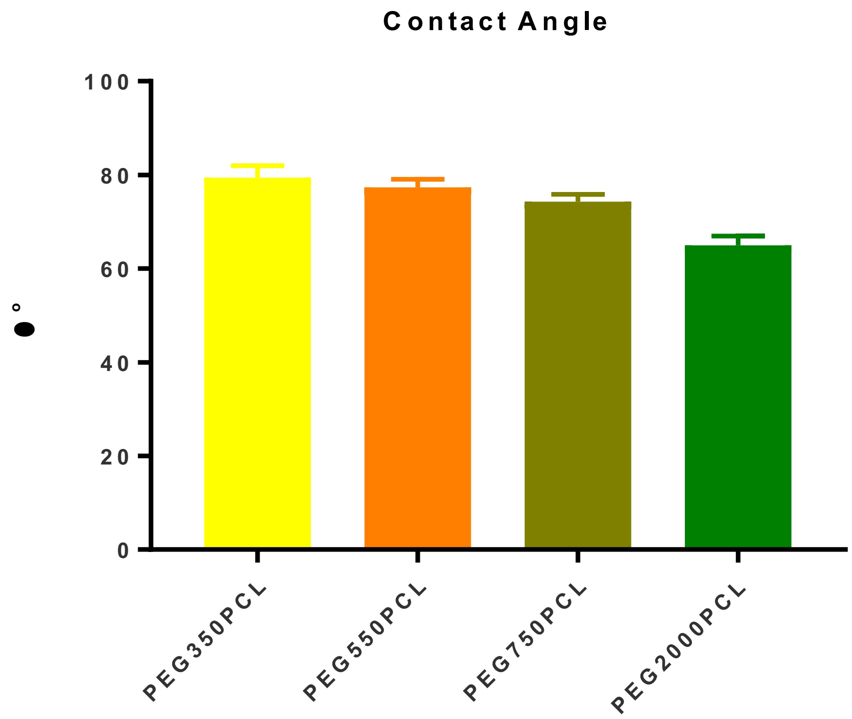

SI Figure 22: Contact angle measurements of the 4 diblock copolymers for the assessment of their wettability properties. Error bars correspond to the standard deviation calculated from four measurements. The θº values decrease with the increase of the hydrophilic block reflecting the increased hydrophilicity of the polymers.

Polymer Characterisation: DSC

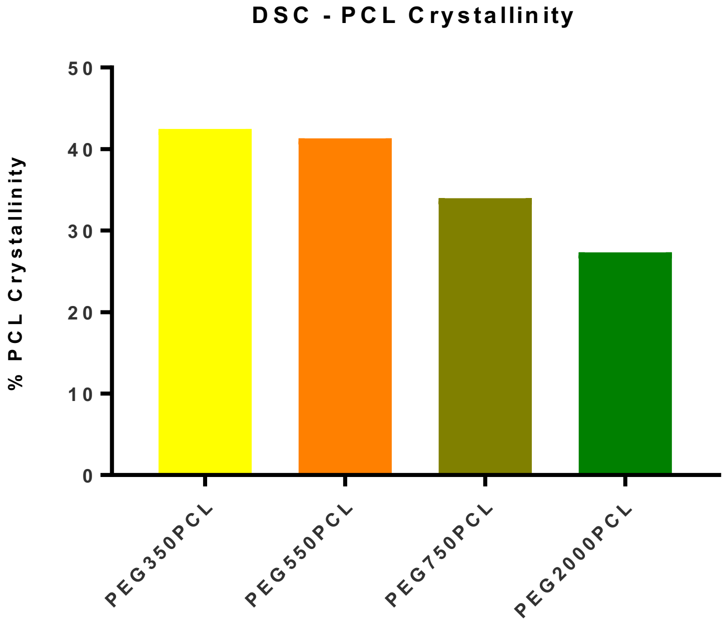

SI Figure 23: Analysis of ΔH measurements from DSC revealed a decrease in the percentage of crystallinity of the PCL block of the copolymers with an increase of the length of the hydrophilic block.

The contribution of the mPEG chain in the ΔH of the copolymers was calculated by: $\Delta H_{mPEG} = \Delta H * \left(\frac{w}{w}\%(mPEG)\right) = \Delta H * \frac{Mw_{mPEG}}{114.14*DP_{PCL}+Mw_{mPEG}}$ where DP_{PCL} is the number of CL units in the PCL blocks as calculated via H^1 NMR. Then Δ_{HPCL}=ΔH-ΔH_{mPEG}.

Polymeric NP Characterisation: DLS

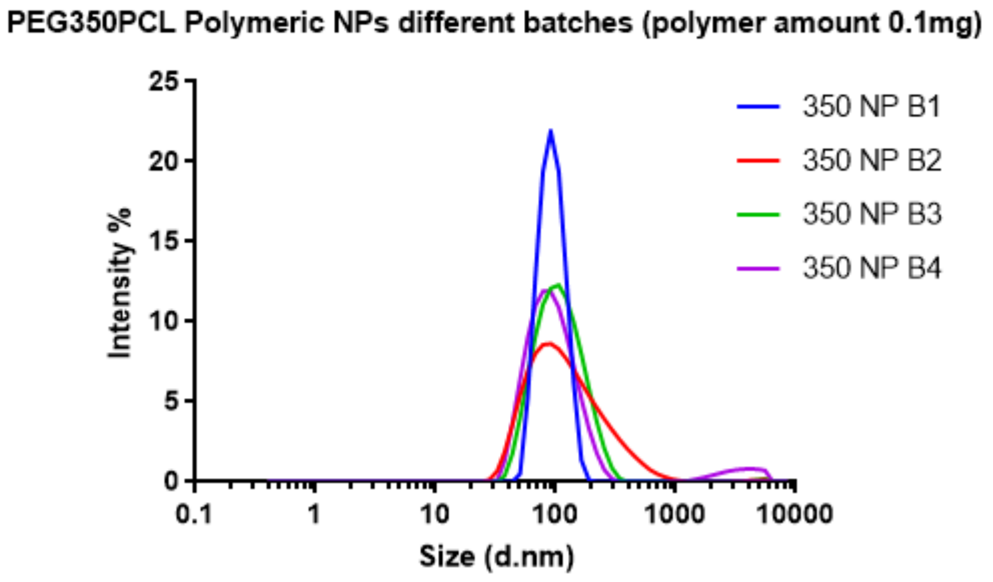

SI Figure 24: Reproducibility of the interfacial deposition method; Overlay of size measurements (DLS) of 4 batches of polymer NPs formed with PEG350PCL at a 0.1 mg polymer amount.

Polymeric NP Characterisation: ζ-potential

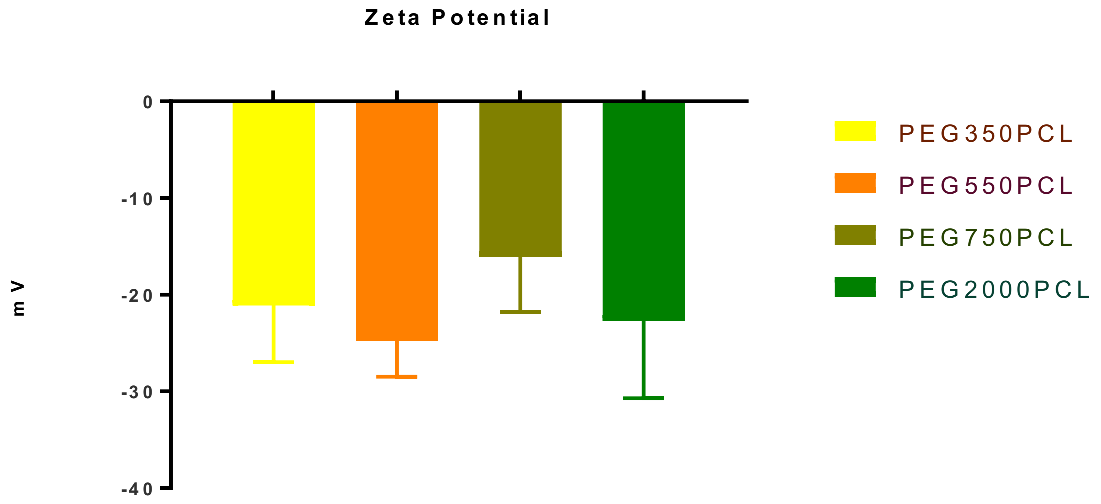

SI Figure 25: Zeta potential of the polymeric NPs. Key refers to PEG chain length of polymer.

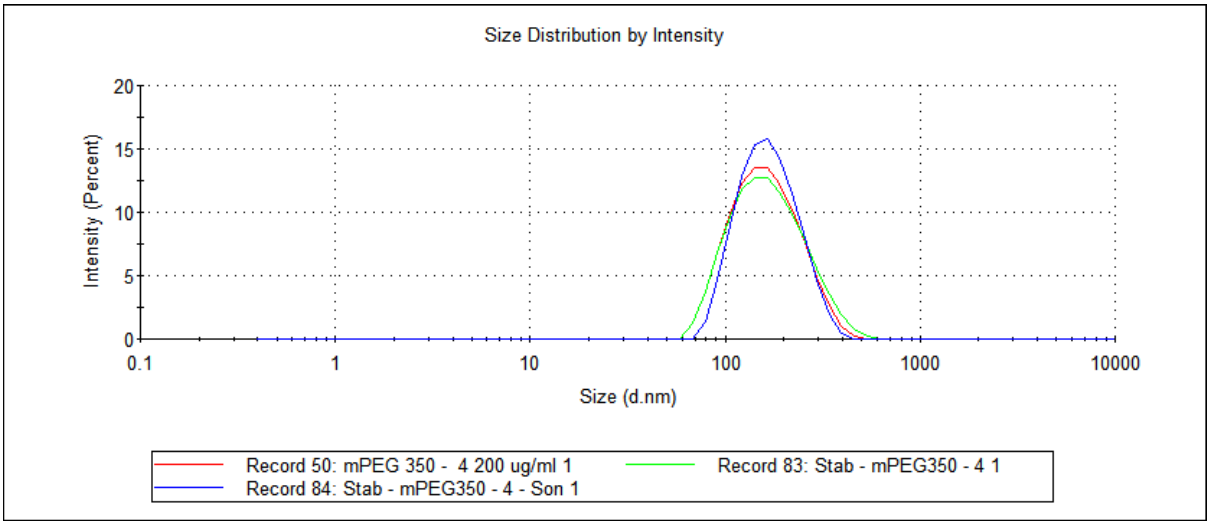

SI Figure 26: Stability of the PEG350PCL polymeric NPs (final polymer concentration in water 0.2 mg/ml): The original measurement is in red, the same sample after 1 month in green and the blue is after sonication of the latter for 5 minutes. The size distribution is the same in all 3 cases.

Polymeric NP Characterisation TEM

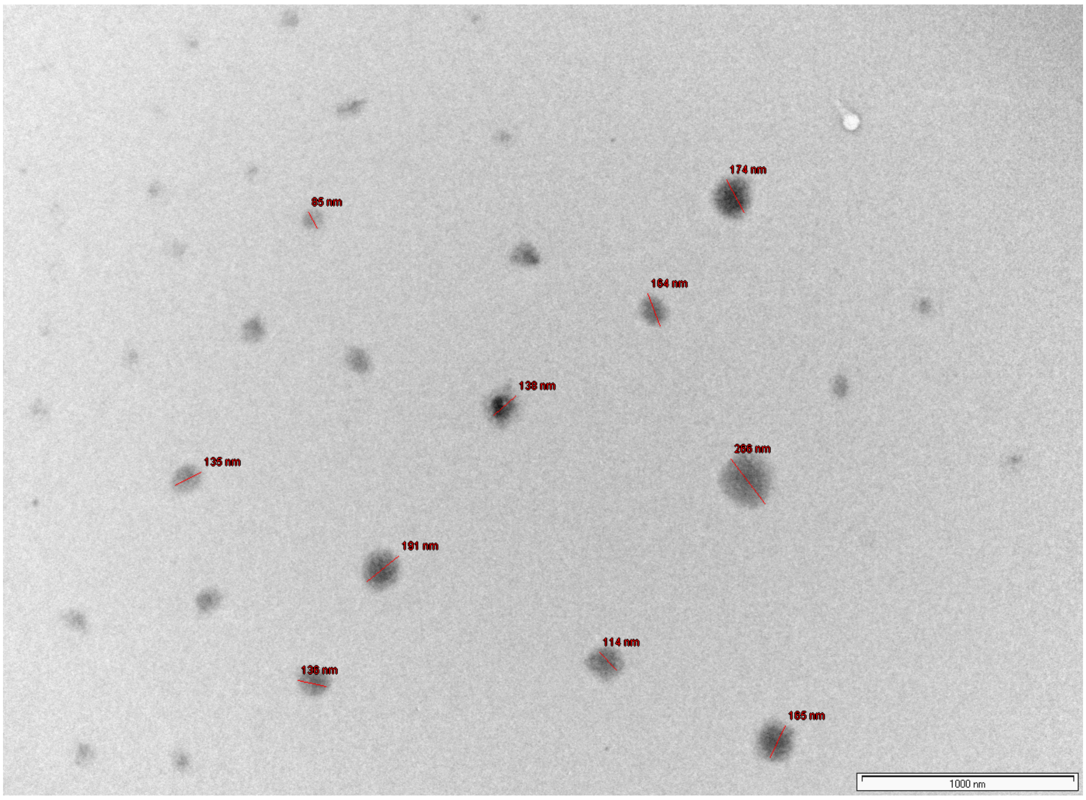

SI Figure 27: TEM image of mPEG350- PCL 2000 polymeric NPs at 0.05 mg/ml initial concentration in acetone (polymer amount 1mg). Diameters of selected NP indicated. Scale bar 1000 nm.

Indomethacin bulk characterisation: DSC

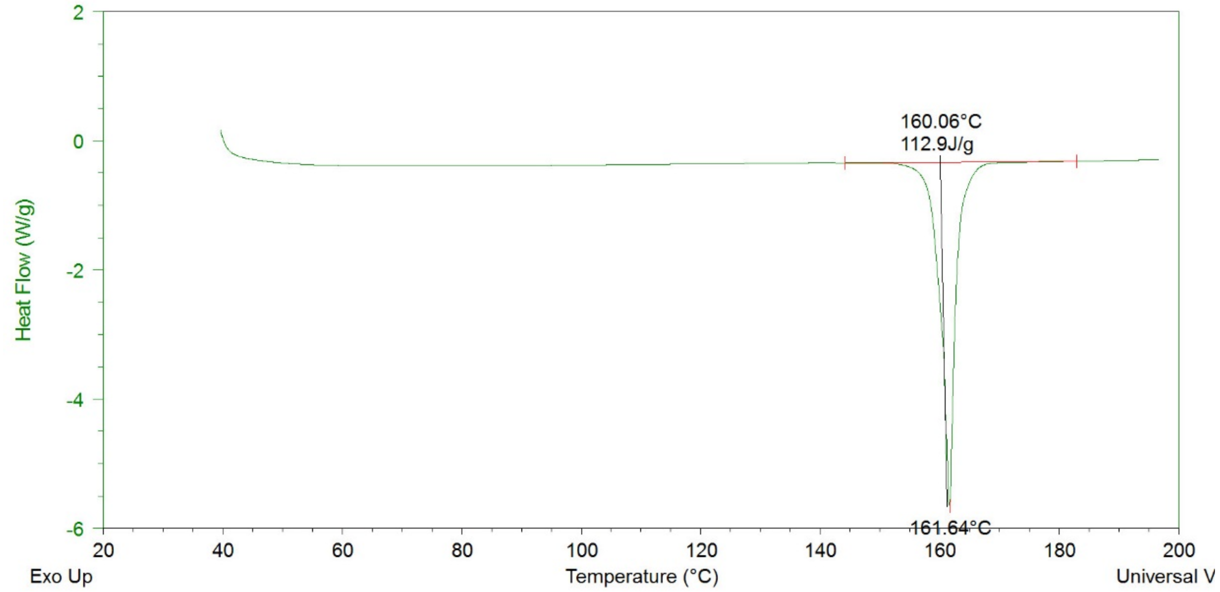

SI Figure 28: DSC of indomethacin as received

Indomethacin NP Characterisation: DLS

			Size (d.nm):	% Intensity:	St Dev (d.nm):
Z-Average (d.nm):	687.2	Peak 1:	712.2	96.1	357.0
PdI:	0.377	Peak 2:	5199	3.9	526.2
Intercept:	0.836	Peak 3:	0.000	0.0	0.000
Result quality :	Good				

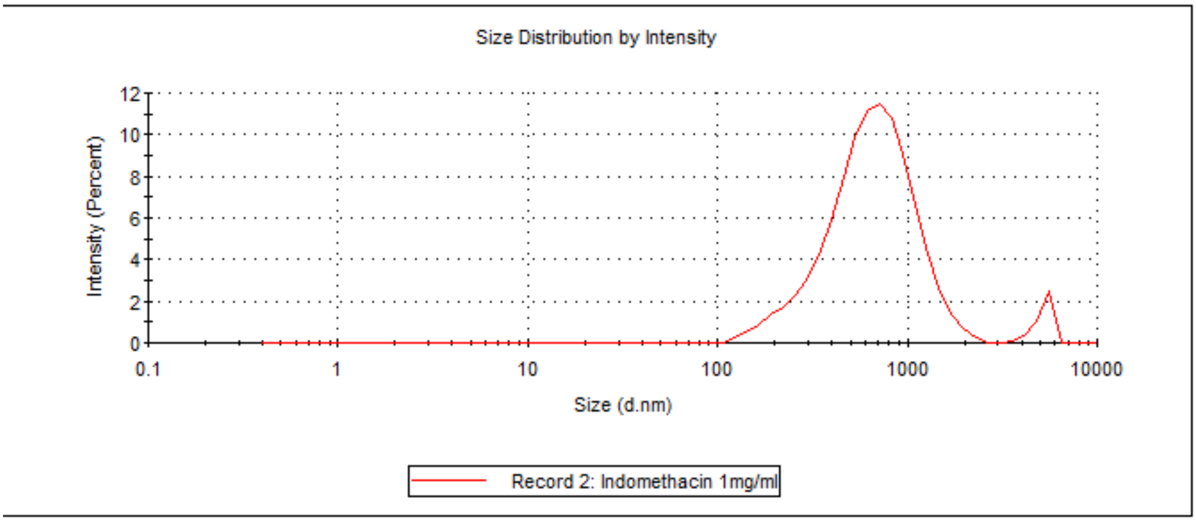

SI Figure 29: Size distribution of Indomethacin in water (1mg/ml), just after sonication, by DLS.

Indomethacin NP Characterisation: POM

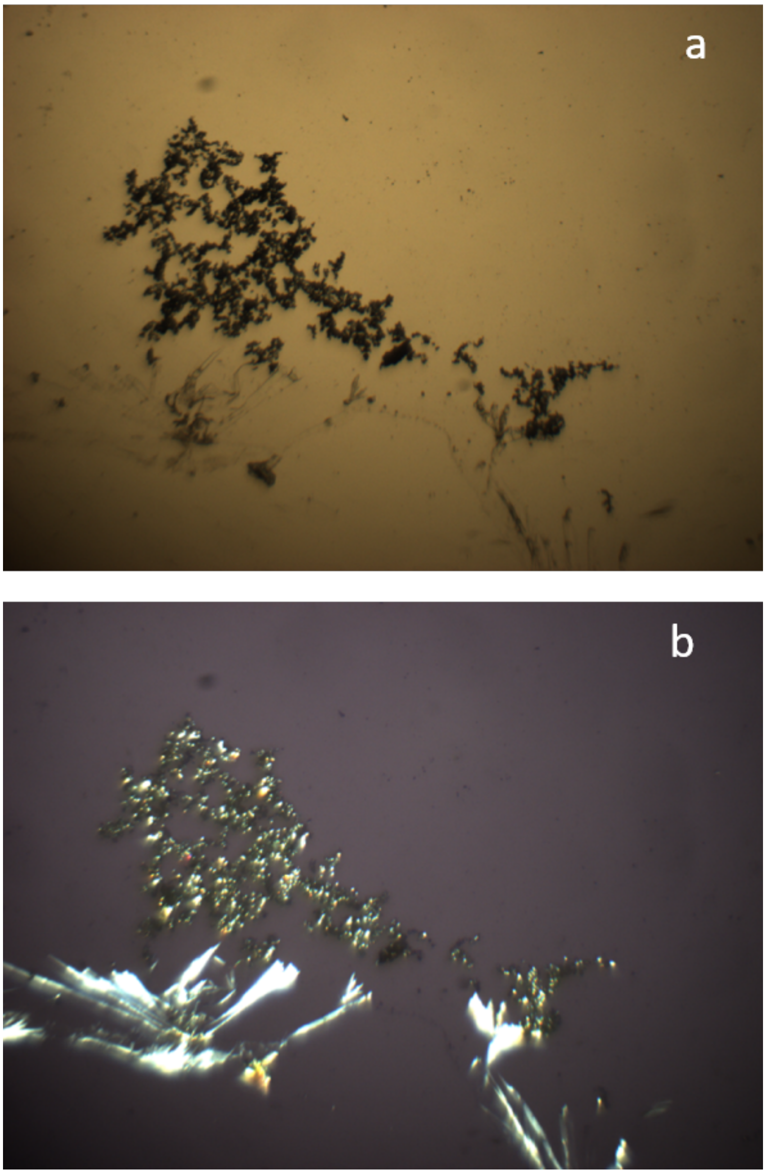

SI Figure 30: Polarised optical microscopy images of indomethacin in aqueous phase prior to the coating experiments. (a) without and (b) with the polariser. Indomethacin is clearly crystalline. Magnification 10x.

Polymer-coated indomethacin NP Characterisation: DLS

			Size (d.nm):	% Intensity:	St Dev (d.nm):
Z-Average (d.nm):	217.3	Peak 1:	252.1	100.0	103.7
PdI:	0.141	Peak 2:	0.000	0.0	0.000
Intercept:	0.919	Peak 3:	0.000	0.0	0.000
Result quality :	Good				

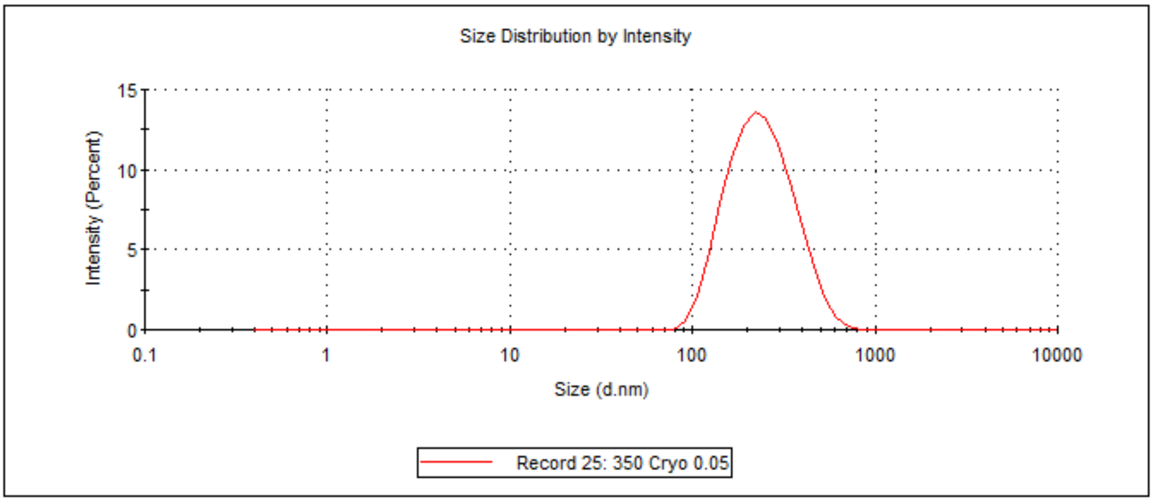

SI Figure 31: mPEG350-PCL2000 coated indomethacin nanoparticle size distribution by DLS. (polymer amount 0.1mg)

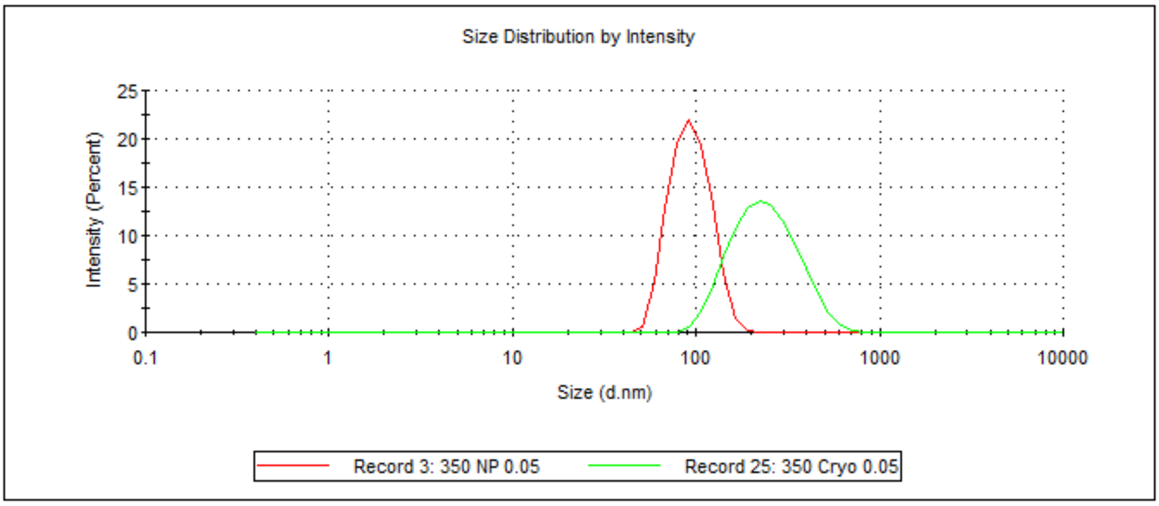

SI Figure 32: Overlay of two experiments with mPEG350-PCL2000 and mPEG350-PCL2000 coated indomethacin nanoparticles (0.1mg polymer amount) to demonstrate the difference between the populations: Red line corresponds to the polymeric NPs that are formed in the absence of indomethacin, while the green distribution relates to the polymer-coated indomethacin

	Size (d.nm):	% Intensity:	St Dev (d.nm):
Z-Average (d.nm): 308.0			
Peak 1:	265.5	96.5	64.89
Peak 2:	5319	3.5	402.5
Peak 3:	0.000	0.0	0.000

PdI: 0.324

Intercept: 0.906

Result quality : Good

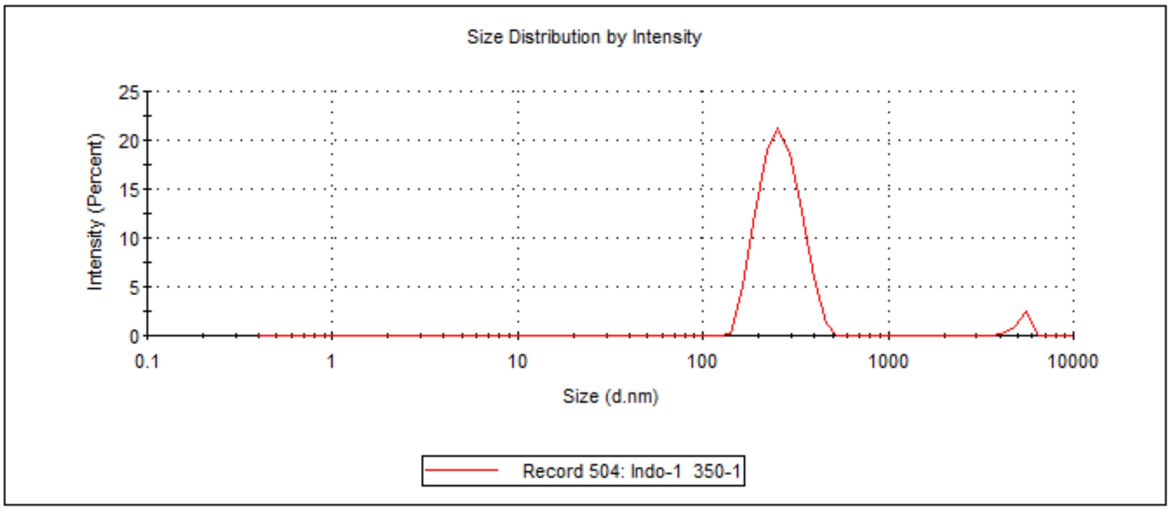

	Size (d.nm):	% Intensity:	St Dev (d.nm):
Z-Average (d.nm): 240.0			
Peak 1:	260.5	100.0	77.99
Peak 2:	0.000	0.0	0.000
Peak 3:	0.000	0.0	0.000

PdI: 0.116

Intercept: 0.916

Result quality : Good

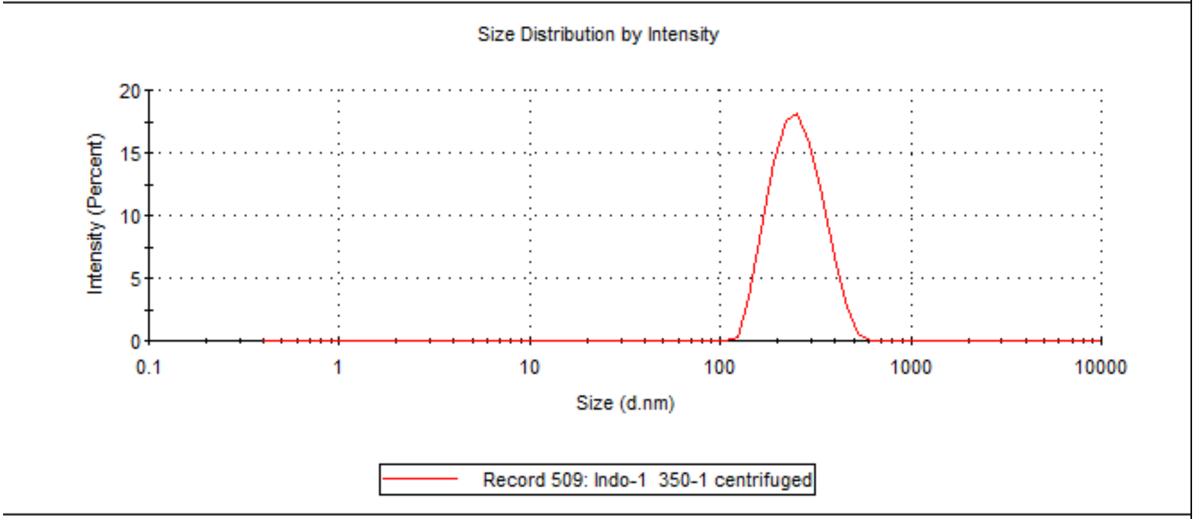

SI Figure 33: Centrifugation of the polymer coated drug nanoparticles (bottom graph) gives a PDI of 0.116, compared to a PDI that ranged from 0.394-0.265 in the pre-centrifugation suspensions (top graph)

Polymer-coated indomethacin NP Characterisation: POM

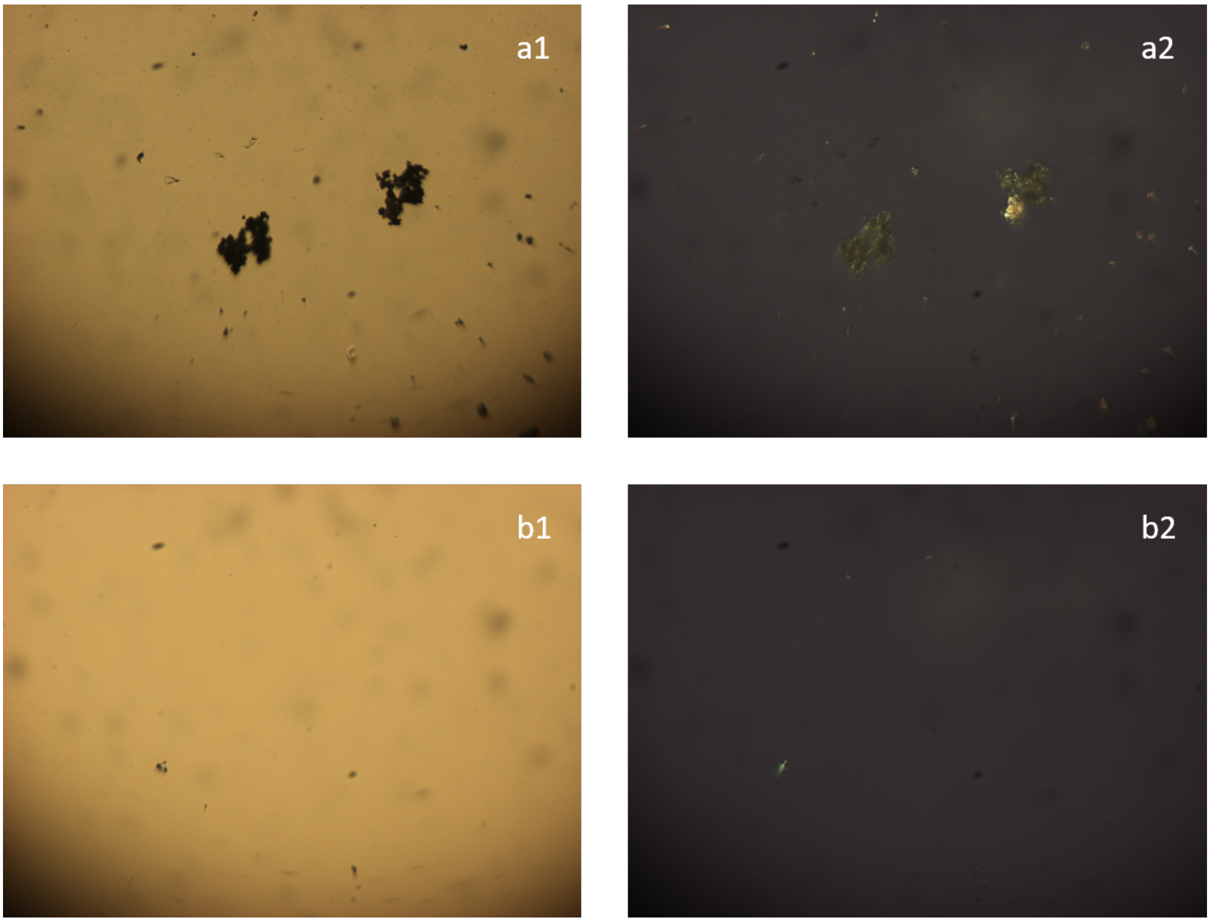

SI Figure 34: Polarised optical microscopy images of the mPEG350PCL-coated indomethacin NPs. (a1) and (a2) prior to centrifugation, where uncoated drug particles form aggregates and (b1), (b2) after the purification where no crystals were observed (Magnification 10x).

Polymer-coated indomethacin NP Characterisation: TEM

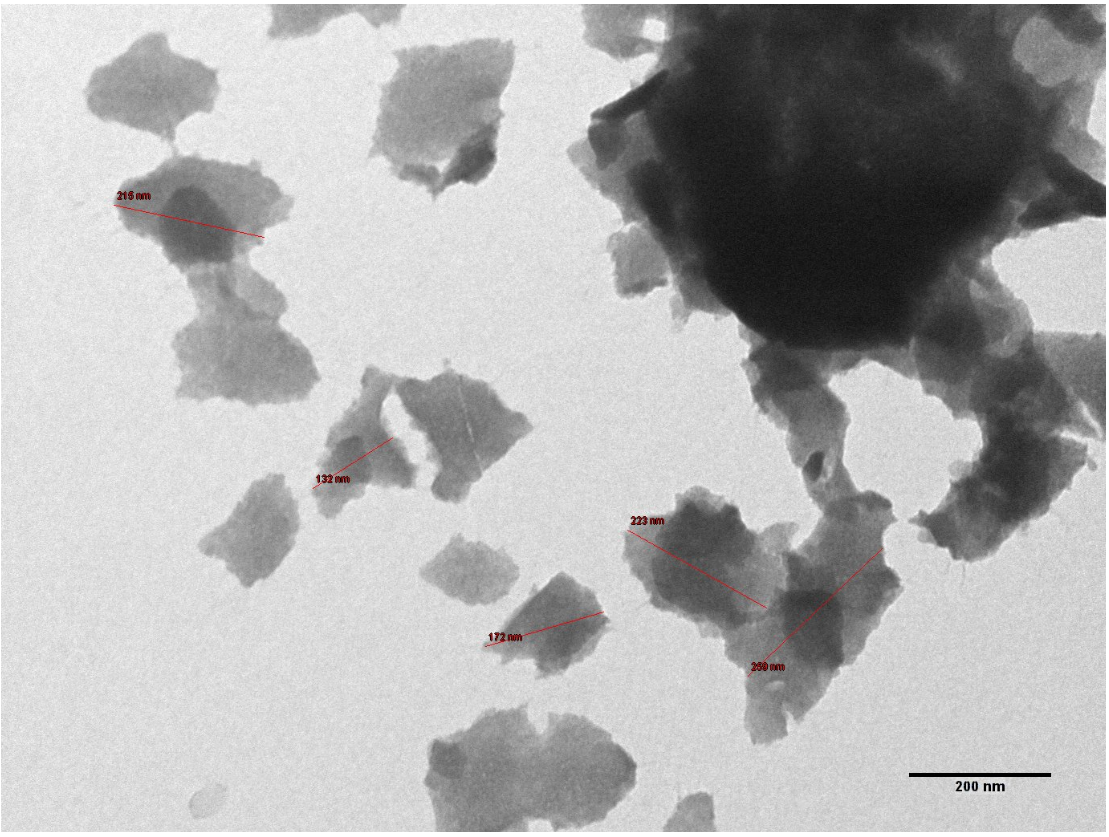

SI Figure 35: TEM picture of mPEG-PCL coated indomethacin particles, (highest polymer starting concentration). Scale bar 200nm.

Polymer-coated indomethacin NP Characterisation: Reproducibility

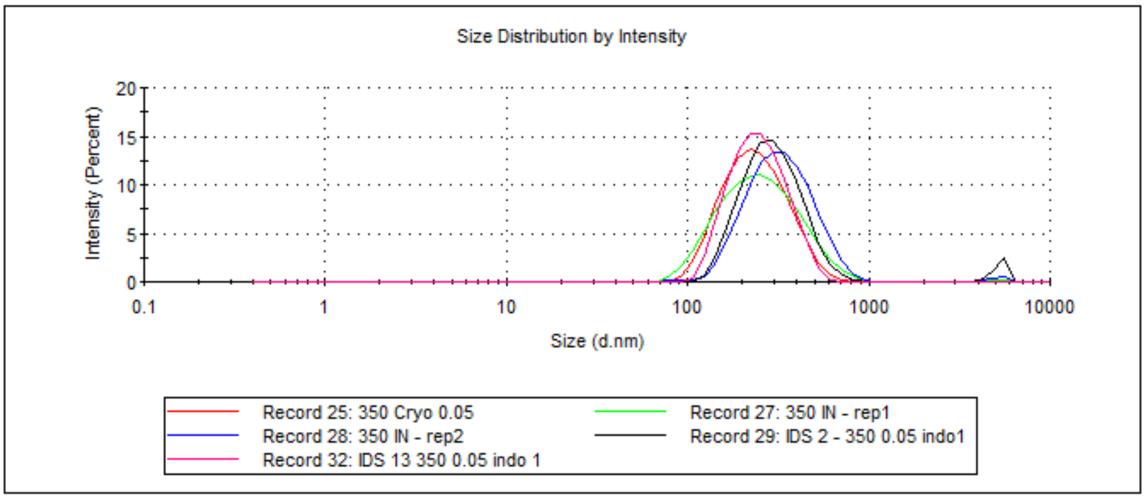

SI Figure 36: Reproducibility of the coating method; Overlay of 5 batches of mPEG350PCL-coated indomethacin NPs (0.1 mg polymer amount, 1 mg/ml indomethacin concentration in water)

Polymer-coated indomethacin NP Characterisation: Stability

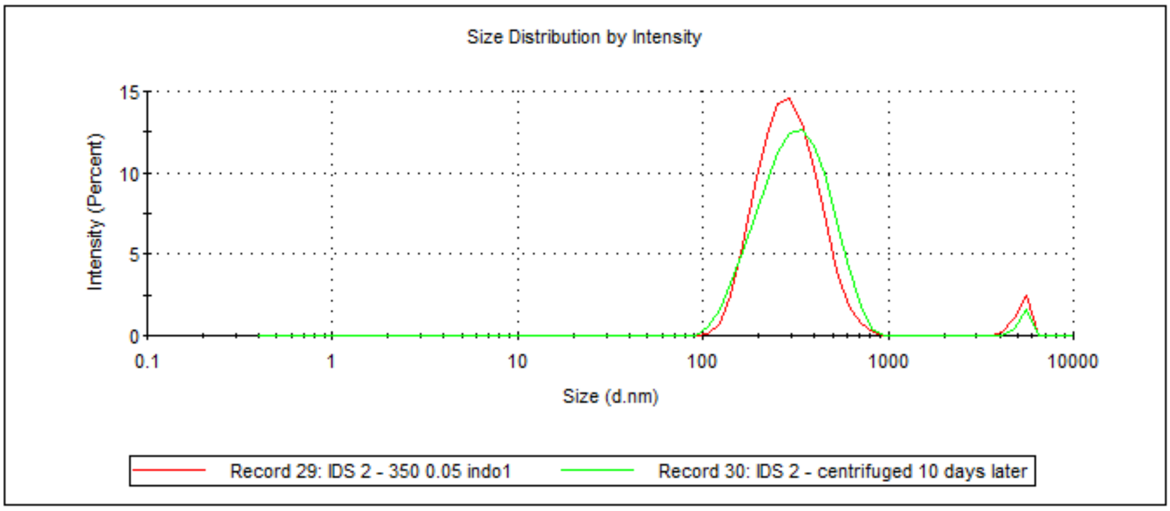

SI Figure 37: Stability of the produced polymer coated-drug NPs; Same batch of mPEG350PCL-coated indomethacin NPs (0.1 mg polymer amount, 1 mg/ml indomethacin concentration in water) measured after 10 days.

Polymer-coated indomethacin NP Characterisation: Effect of Drug amount

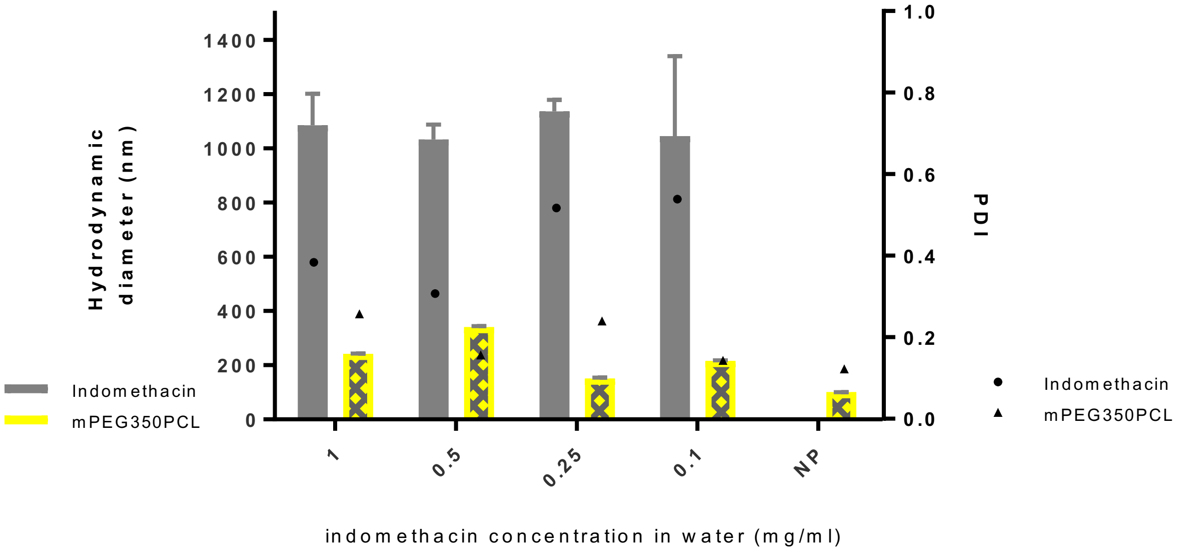

SI Figure 38: Analysis of the effect of decreasing indomethacin concentration in the starting drug-bearing aqueous phase. Y-axis:Size distribution (Intensity), Right Y-axis PDI of measurements.

Polymer-coated indomethacin NP Characterisation: Drug Loading

Table 1 - Calculation of drug loading and encapsulation efficiency via UV-Vis, after lyophilisation of the suspension

mPEG350PCL amount (mg/ml)	Starting Drug amount (mg)	% Drug loading	Entrapment efficiency (eq. 2)	Entrapment efficiency ‡ (amended eq. 2)
1	1	13.79	16.0	95.53
0.5	1	23.17	15.32	91.47
0.1	1	64.39	18.41	-
0.05	1	78.27	18.31	99.02

% Drug loading = polymer amount/ (polymer amount + drug amount in nanoparticles) *100
(Assuming all polymer is still present in drug coated nanoparticles)

In order to account for the drug loss during the loading of the aqueous bearing syringe and the pumping, Ep2 was amended into the following:
%Entrapment Efficiency ‡
= (amount of coated drug/amount of recovered control drug NPs)*100.
